# Electrochemically‐Switched 2nd Order Non‐Linear Optical Response in an Arylimido‐Polyoxometalate with High Contrast and Cyclability

**DOI:** 10.1002/anie.202215537

**Published:** 2022-12-27

**Authors:** Bethany R. Hood, Yovan de Coene, Afonso V. Torre Do Vale Froes, Claire F. Jones, Pierre Beaujean, Vincent Liégeois, Fraser MacMillan, Benoît Champagne, Koen Clays, John Fielden

**Affiliations:** ^1^ School of Chemistry University of EastAnglia Norwich NR4 7TJ UK; ^2^ Department of Chemistry University of Leuven Celestijnenlaan 200D 3001 Leuven Belgium; ^3^ Laboratory of Theoretical Chemistry Unit of Theoretical and Structural Physical Chemistry NISM (Namur Institute of Structured Matter) University of Namur Rue de Bruxelles, 61 5000 Namur Belgium

**Keywords:** Donor-Acceptor Systems, Molecular Devices, Nonlinear Optics, Polyoxometalates, Spectroelectrochemistry

## Abstract

Electrochemically switched 2^nd^ order non‐linear optical responses have been demonstrated for the first time in polyoxometalates (POMs), with an arylimido‐derivative showing a leading combination of high on/off contrast (94 %), high visible transparency, and cyclability. Spectro‐electrochemical and TD‐DFT studies indicate that the switch‐off results from weakened charge transfer (CT) character of the electronic transitions in the reduced state. This represents the first study of an imido‐POM reduced state, and demonstrates the potential of POM hybrids as electrochemically activated molecular switches.

## Graphical abstract

Charge transfer transitions in a new arylimido‐polyoxometalate (POM) are turned off by reduction, enabling a high contrast (94 %) electrochemically switched 2^nd^ order non‐linear optical (NLO) response. The overall combination of on/off contrast, high visible transparency and multicycle stability is leading in switchable 2^nd^ order NLO chromophores, and shows for the first time the strong potential of POM hybrids as redox‐driven optical switches.

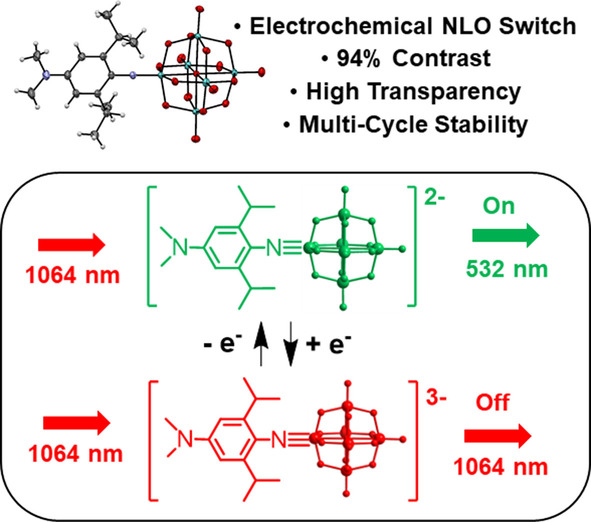

Since their first observation in the 1960s,^[1]^ non‐linear optical (NLO) effects have become essential to current and future technologies involving generation and manipulation of laser light: for example telecommunications, optical and electro‐optical computing, optical data storage and biological imaging.^[2]^ For advanced applications in telecommunications and computing, the fast 2^nd^ order NLO responses of molecular charge transfer chromophores promise advantages over traditional inorganic salts, and have led to development of many organic and organometallic NLO chromophores.[^2^, ^3^, ^4^] Molecular 2^nd^ order NLO performance is assessed by measurement of the first hyperpolarizability β , with static (zero‐frequency) values $\beta_{0}$ used to facilitate comparison. Yet, while materials with extremely high $\beta_{0}$ values (≫10^−27^ esu) have been obtained,^[5]^ only dimethylaminostilbazolium tosylate (DAST), based on the DAS^+^ cation ($\beta_{0}$ =25×10^−30^ esu),[^3a^, ^3b^] is in current technological use.

Two key challenges for molecular NLO materials are (i) developing chromophores that combine high activity with adequate transparency,^[6]^ and (ii) producing materials whose properties can be switched.[^7^, ^8^, ^9^] Regarding (i), structural modifications that increase β usually involve stronger electron donor/acceptor pairs or extended π‐systems that increase conjugation—these lower HOMO–LUMO gaps and thus tend to increase absorption of visible and near‐infrared light, leading to lower device efficiency and reduced photostability. For (ii), most molecular NLO switches have relied on redox reagents or other chemical stimuli (pH) which are impractical for application in devices,^[7]^ or photoisomerization^[8]^ which due to structural change is slower and more vulnerable to side reactions than electron transfer. Electrochemical oxidation of a donor or reduction of an acceptor potentially provides a rapid and device compatible means of switching NLO responses by turning on and off charge transfer transitions,^[9]^ with less risk of degradation. However, the few existing examples are all based on materials with strong low energy metal‐to‐ligand charge transfer transitions (MLCT), whose activity is turned off by oxidizing the metal center. Reabsorption of second harmonic light by such MLCT transitions is intrinsically problematic for eventual construction of stable, efficient molecule‐based NLO switches.

Herein, we describe a new strategy based on electrochemical switching of higher energy ligand‐to‐polyoxometalate CT (LPCT) transitions. Polyoxometalates (POMs) are well known for fast, stable redox chemistry, as they accept electrons with almost no structural change.^[10]^ Moreover, owing to push‐pull CT effects, we previously demonstrated that NLO chromophores based on arylimido‐derivatized Lindqvist POM acceptors ([Mo_6_O_18_NAr]^2−^) show high static first hyperpolarizabilities $\beta_{0}$ , combined with high transparency, giving better transparency/non‐linearity trade‐offs than typical purely organic systems.^[11]^ Here, introducing bulky groups (iPr) around the Mo=N bond has enabled us to stabilize and characterise the [Mo_6_O_18_NAr]^3−^ state for the first time, revealing the strongest overall performance of any switchable NLO chromophore to date: *β*_0_,_zzz_=89×10^−30^ esu (by hyper‐Rayleigh Scattering, HRS), high transparency beyond 500 nm, 94 % on/off contrast, and the best cyclability yet demonstrated—4 complete cycles in electrochemistry‐HRS, 16 by linear spectroelectrochemistry, with minimal degradation.

Donor‐acceptor POM derivative [Mo_6_O_18_NPh(^i^Pr)_2_NMe_2_]^2−^ ([**1**]^2−^, Figure 1) was accessed as a tetrabutylammonium salt following established methods,[^11^, ^12^, ^13^] and thoroughly characterized (see Supporting Information, Scheme S1). X‐ray quality crystals of [NBu_4_]_2_[**1**]⋅Me_2_CO were obtained, enabling acquisition of a high‐quality crystal structure (Figure 2, Table S1).^[14]^ The Mo−O bond lengths of the cluster are comparable to other hexamolybdate derivatives (Table S2),[^13a^, ^15^] while steric repulsion and C−H…O hydrogen bonds (C(H)…O distances ca. 3.3 to 3.7 Å)^[16]^ between the POM core and the isopropyl groups result in a similar length but more linear imido bond (1.741(1) Å, 175.0(2)°) than observed in other POM‐based chromophores.^[11]^ The slightly less quinoidal structure of the ligand in [**1**]^2−^ compared to 2,6‐H analogue [**1H**]^2−^ (Table S3) suggests slightly weakened donor‐acceptor communication.

**Figure 1:**
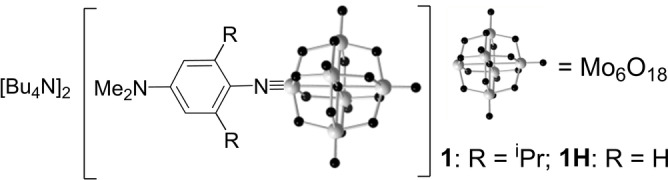
Structures of switchable NLO chromophore [**1**]^2−^, and isopropyl‐free analogue [**1H**]^2−^. [NBu_4_]_2_[**1H**] was previously published.^[11b]^

**Figure 2:**
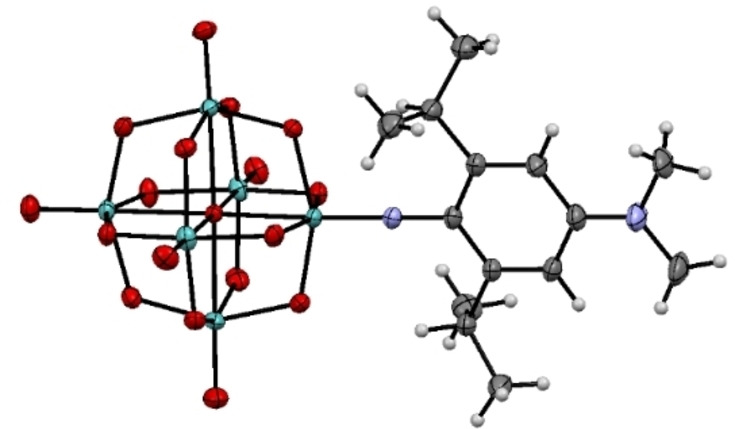
ORTEP representation of the crystal structure of the complex anion [**1**]^2−^. C, is grey; N, purple; O, red; and Mo, green, H atoms are white circles with arbitrary radii. Thermal ellipsoids are at the 30 % probability level.

Characterization of [NBu_4_]_2_[**1**] by cyclic voltammetry (CV, Figure S2) finds a [**1**]^2−/3−^ reduction peak with near ideal reversibility (Table 1), negatively shifted by *ca*. 60 mV from that previously reported for [**1H**]^2−/3−^.^[11b]^ This indicates a more electron rich POM cluster, resulting from inductive donation from the ^i^Pr groups ortho to the imido group. A second, irreversible reduction is observed at −1.92 V vs Fc/Fc^+^, while quasi‐reversible oxidation of the amine was seen at 0.30 V vs Fc/Fc^+^. UV/Vis spectroscopy reveals a ligand‐to‐POM (LPCT) charge transfer peak at 431 nm (Figure 3, Table 1), slightly redshifted from [**1H**]^2−^ (424 nm). This is ascribed to inductive donation from the ^i^Pr groups raising the level of the HOMO, which DFT calculations (ωB97X‐D/6‐311G(d)/LanL2TZ, MeCN solvation by IEFPCM) indicate is spread across the entire arylimido moiety (Figure 4a), more than the POM based LUMO. TD‐DFT calculations reproduce the LPCT peak well, although the (vertical excitation) energy is over‐estimated slightly (0.3 eV) vs experiment (Figure S4). The LPCT character of the lowest‐energy band is stronger for the oxidised than the reduced form, as shown by the excitation‐induced electron density differences (Figure S5). Subsequently, the change of dipole moment upon excitation is slightly smaller for the reduced form than the oxidised one (8.0 versus 9.6 D).

**Table 1:** UV/Vis Absorption and Electrochemical Data for [**1**]^2−^ and [**1**]^3−^ in MeCN.

Anion	*λ*_max_/nm^[a]^ (*ϵ*, 10^3^ M^−1^ cm^−1^)	*E*_max_/eV	*E*/V *vs* Fc/Fc^+^ (Δ*E*_p_/mV)^[b]^		
			*Epc* [**1**]^3−/4−^	*E*_1/2_ [**1**]^2−/3−^	*E*_1/2_ [**1**]^1−/2−^
[**1**]^2−^	206 (44.3) 258 (28.1) 431 (30.7)	6.02 4.81 2.88	−1.92	−1.10 (73)	0.30 (73)
[**1**]^3−^	200 (53.4) 256 (29.4) 398 (25.6)	6.20 4.84 3.12			

[a] Data for [**1**]^2−^ obtained at ca. 10^−5^ M in MeCN. Data for [**1**]^3−^ obtained at ca. 10^−2^ M in 0.3 M [NBu_4_][BF_4_] using a thin layer spectroelectrochemistry cell, with initial [**1**]^2−^ absorbances as a reference. [b] Solutions *ca*. 10^−3^ M in analyte, 0.1 M in [NBu_4_][BF_4_] at a glassy carbon working electrode, scan rate 100 mV s^−1^.

**Figure 3:**
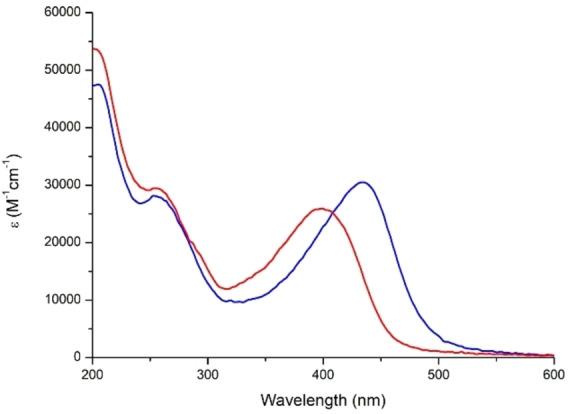
UV/Vis spectra of [**1**]^2−^ (blue) and [**1**]^3−^ (red) obtained by spectroelectrochemistry (electrolyte 0.3 M NBu_4_BF_4_ in MeCN). 98 % recovery of the original spectrum was seen upon re‐oxidation.

**Figure 4:**
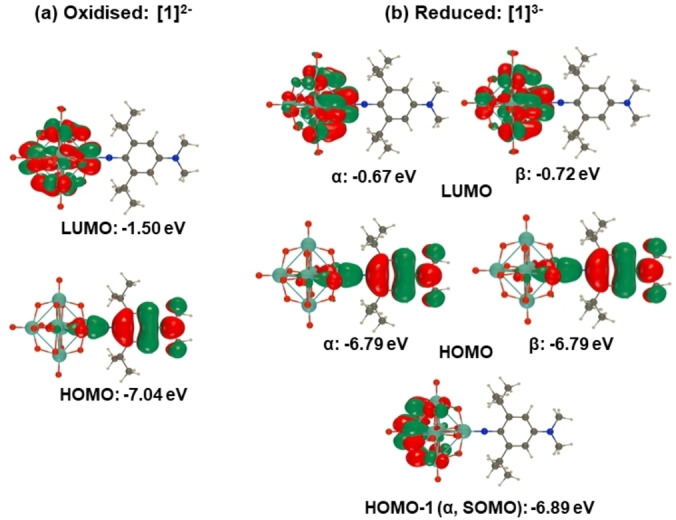
DFT computed frontier orbitals for [**1**]^2−^ and reduced state [**1**]^3−^, at the ωB97X‐D/6‐311G(d)/LanL2TZ level of theory.

To probe the stability of the [**1**]^3−^ reduced state, and its suitability for redox‐switched HRS, we investigated the first reduction of [**1**]^2−^ by bulk electrolysis. Investigation of unprotected derivatives such as [**1H**]^2−^ in similar conditions has indicated solvolysis of the reduced state leads to loss of ligand and conversion of ≥80 % of the derivative into [Mo_6_O_19_]^2−/3−^.^[17]^ However, for sterically protected [NBu_4_]_2_[**1**], CV peak currents measured after 20 minutes reduction at −1.2 V vs Fc/Fc^+^ indicate minimal loss of the imido compound (Figure S3). Subsequent spectroelectrochemical investigation showed that upon reduction the low energy *λ*_max_ blue shifts 33 nm (0.24 eV), to 398 nm, and loses intensity (Figure 3) as the POM acceptor weakens—DFT calculated LUMOs are ca. 0.8 eV higher in energy for the reduced state, consistent with the electrochemically observed spacing of *ca*. 0.8 V between first and second reductions. TD‐DFT calculations fit these observations, showing a blue shift (24 nm, 0.20 eV), and reduction of the intensity, accompanying a lessened LPCT character of the transition (Figures S4/S5). Re‐oxidation of the POM core resulted in a 98 % recovery of the original sample based on the intensity of the LPCT peak, confirming the stability of the reduced state and the prospects for reversible switching. To further characterize the [**1**]^3−^ state we performed X‐band EPR. At 67 K, this reveals a very similar signal to that of [Mo_6_O_19_]^3−^, where an absence of measurable hyperfine coupling indicates delocalization of the electron across the POM cluster. At lower temperatures (10 K), clear evidence of coupling to a single Mo centre, but no large N hyperfine couplings, could be observed (Figure S7). This is consistent with DFT calculations showing a SOMO (slightly stabilized over the α and β HOMOs) based predominantly on the Mo opposite the imido group (Figure 4b), and is logical in showing the electron localizing away from the imido‐donor.

Hyper‐Rayleigh scattering (HRS) performed at 1064 nm revealed a static first hyperpolarizability $\beta_{0,zzz}$ =89×10^−30^ esu for [NBu_4_]_2_[**1**]—almost identical to that previously obtained for [NBu_4_]_2_[**1H**] (Table 2).^[11b]^ Thus [**1**]^2−^, like previous “POMophores”, shows a better combination of transparency and activity than typical purely organic systems—and much better than existing redox switched NLO chromophores, whose activity is based on low energy (*λ*_max_>500 nm) MLCT transitions.

**Table 2:** Comparison of experimental and computational values of hyperpolarizability for [**1**]^2−^ and its reduced state [**1**]^3−^

	*β*_1064,HRS_^[a]^	*β*_1064,zzz_^[a]^	*β*_0,zzz_^[a]^	Computed β_1064,HRS_^[b]^
[**1**]^2−^	127.9	309.0	88.8	149.0
[**1**]^3−^	8.3	20.0	6.7	57.9

[a] Experimental values, ×10^−30^ esu, $\beta_{0,zzz}$ calculated from the dynamic hyperpolarizability and the experimental λ_max_ using the two‐state model. [b] computational values, ×10^−30^ esu, after correcting for pre‐resonance effects and finite bandwith of the UV/Visible absorption band (see Supporting Information).

A specially designed electrochemical cell (Figure S8, SI) enabled coupled electrochemistry/HRS measurements, which showed a near 94 % decrease in second harmonic signal upon reduction of [**1**]^2−^, and a $\beta_{0,zzz}$ of only 7×10^−30^ esu for the [**1**]^3−^ reduced state—a very high contrast switching effect (Figures 5, S9). Reoxidation recovered 90 % of the initial signal, which was retained over a further three off/on cycles. The smaller switch‐off in later cycles is likely a result of potential drift, as chronoamperometry data reveal less charge was passed and so less complete (ca. 92 %) reduction was achieved. The dispersion corrected TD‐DFT computed dynamic $\beta_{1064,HRS}\;$ value, of 149×10^−30^ esu for [**1**]^2−^, is a good match for experiment, the calculated reduced state β is a large overestimate, but does reveal an attenuation consistent with the experimental trend. Stability over more cycles has been demonstrated by spectroelectrochemistry, (Figure S10) which shows 95 % retention of the absorbance at *λ*_max_ after 16 cycles. Almost all of the decline occurs in the first 8 cycles, with the absorbance remaining almost constant afterwards. The improved stability in later scans may result from consumption of adventitious water in the first cycles, presence of decomposition products shifting equilibria in favour of [**1**]^2−/3−^, and also reflects establishment of equilibria between [**1**]^2−^ and [**1**]^3−^ after the initial cycles, which may not be completely overcome electrochemically. In any case, the results show that [**1**]^2−^ is an electrochemically addressable, molecular 2^nd^ order NLO switch with very high contrast and leading multi‐cycle stability.^[18]^

**Figure 5:**
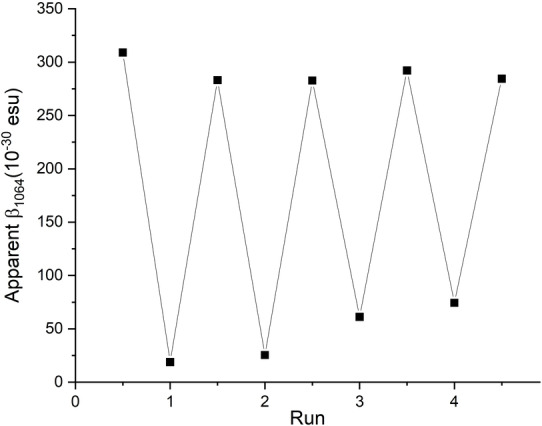
Electrochemically switched HRS signal in [**1**]^2−^ (on) and [**1**]^3−^ (off). The smaller “switch off” in runs 3 and 4 is a result of incomplete reduction.

In conclusion, we have shown for the first time that arylimido‐Lindqvist reduced states can be effectively stabilized by placing sterically bulky groups around the Mo=N bond. Although the reduced state SOMO is located away from the imido group, reduction dramatically weakens CT to the POM, producing a reversible 94 % switch‐off in 2^nd^ order NLO activity. Combined with leading cyclability and transparency, this gives [**1**]^2−^ the best overall performance of any molecular NLO switch to date. Future work will focus on translating these results into thin films,^[9b]^ to produce bulk materials capable of electrochemical‐NLO switching. More broadly, the work highlights the strong potential of imido‐POM hybrids as molecular switches, which we are continuing to explore in new structures.

## Conflict of interest

The authors declare no conflict of interest.

## Supporting information

As a service to our authors and readers, this journal provides supporting information supplied by the authors. Such materials are peer reviewed and may be re‐organized for online delivery, but are not copy‐edited or typeset. Technical support issues arising from supporting information (other than missing files) should be addressed to the authors.

Supporting Information

Supporting Information

Supporting Information

## Data Availability

The data that support the findings of this study are available from the corresponding author upon reasonable request.

## References

[1] P. A. Franken , A. E. Hill , C. W. Peters , G. Weinreich , Phys. Rev. Lett. 1961, 7, 118.

[2a] Nonlinear Optics of Organic Molecules and Polymers (Eds.: H. S. Nalwa , S. Miyata ), CRC Press, Boca Raton, FL, 1997;

[2b] S. R. Marder , Chem. Commun. 2006, 131–134; 10.1039/b512646k 16372084

[2c] M. G. Kuzyk , J. Mater. Chem. 2009, 19, 7444;

[2d] M. Pawlicki , H. A. Collins , R. G. Denning , H. L. Anderson , Angew. Chem. Int. Ed. 2009, 48, 3244; 10.1002/anie.200805257 19370705

[3a] S. R. Marder , J. W. Perry , W. P. Schaefer , Science 1989, 245, 626; 17837617 10.1126/science.245.4918.626

[3b] B. J. Coe , J. A. Harris , I. Asselberghs , K. Clays , G. Olbrechts , A. Persoons , J. T. Hupp , R. C. Johnson , S. J. Coles , M. B. Hursthouse , K. Nakatani , Adv. Funct. Mater. 2002, 12, 110;

[3c] B. J. Coe , J. A. Harris , I. Asselberghs , K. Wostyn , K. Clays , A. Persoons , B. S. Brunschwig , S. J. Coles , T. Gelbrich , M. E. Light , M. B. Hursthouse , K. Nakatani , Adv. Funct. Mater. 2003, 13, 347;

[3d] B. J. Coe , J. Fielden , S. P. Foxon , M. Helliwell , I. Asselberghs , K. Clays , K. De Mey , B. S. Brunschwig , J. Org. Chem. 2010, 75, 8550; 21080634 10.1021/jo101966r

[3e] S.-H. Jang , J. Luo , N. M. Tucker , A. Leclercq , E. Zojer , M. A. Haller , T.-D. Kim , J.-W. Kang , K. Firestone , D. Bale , D. Lao , J. B. Benedict , D. Cohen , W. Kaminsky , B. Kahr , J.-L. Brédas , P. Reid , L. R. Dalton , A. K.-Y. Jen , Chem. Mater. 2006, 18, 2982.

[4a] B. J. Coe , J. Fielden , S. P. Foxon , J. A. Harris , M. Helliwell , B. S. Brunschwig , I. Asselberghs , K. Clays , J. Garín , J. Orduna , J. Am. Chem. Soc. 2010, 132, 10498; 20617798 10.1021/ja103289a

[4b] B. J. Coe , J. Fielden , S. P. Foxon , M. Helliwell , B. S. Brunschwig , I. Asselberghs , K. Clays , J. Olesiak , K. Matczyszyn , M. Samoc , J. Phys. Chem. A 2010, 114, 12028; 20977249 10.1021/jp106473e

[4c] L. T. Cheng , W. Tam , S. H. Stevenson , G. R. Meredith , G. Rikken , S. R. Marder , J. Phys. Chem. 1991, 95, 10631;

[4d] L. T. Cheng , W. Tam , S. R. Marder , A. E. Steigman , G. Rikken , C. W. Spangler , J. Phys. Chem. 1991, 95, 10643.

[5a] H. Xu , F. Liu , D. L. Elder , L. E. Johnson , Y. de Coene , K. Clays , B. H. Robinson , L. R. Dalton , Chem. Mater. 2020, 32, 1408;

[5b] H. Xu , L. E. Johnson , Y. de Coene , D. L. Elder , S. R. Hammond , L. R. Dalton , B. H. Robinson , J. Mater. Chem. C 2021, 9, 2721;

[5c] H. Xu , D. L. Elder , L. E. Johnson , Y. de Coene , S. R. Hammond , W. V. Ghinst , K. Clays , L. R. Dalton , B. H. Robinson , Adv. Mater. 2021, 33, 2104174. 10.1002/adma.202104174 34545643

[6a] G. Alcaraz , L. Euzenat , O. Mongin , C. Katan , I. Ledoux , J. Zyss , M. Blanchard-Desce , M. Vaultier , Chem. Commun. 2003, 2766; 10.1039/b308664j 14651095

[6b] H. Kang , P. Zu , Y. Yang , A. Fachetti , T. J. Marks , J. Am. Chem. Soc. 2004, 126, 15974; 15584726 10.1021/ja045043k

[6c] Y. Shi , D. Frattarelli , N. Watanabe , A. Facchetti , E. Cariati , S. Righetto , E. Tordin , C. Zuccaccia , A. Macchioni , S. L. Wegener , C. L. Stern , M. A. Ratner , T. J. Marks , J. Am. Chem. Soc. 2015, 137, 12521; 26360110 10.1021/jacs.5b04636

[6d] L. Beverina , A. Sanguineti , G. Battagliarin , R. Ruffo , D. Roberto , S. Righetto , R. Soave , L. L. Presti , R. Ugo , G. A. Pagani , Chem. Commun. 2011, 47, 292; 10.1039/c0cc01652g 20725681

[6e] O. Maury , H. Le Bozec , Acc. Chem. Res. 2005, 38, 691; 16171312 10.1021/ar020264l

[6f] H. Xiao , H. Yin , X. Zhang , Org. Lett. 2012, 14, 5282. 23035950 10.1021/ol302443z

[7a] B. J. Coe , S. Houbrechts , I. Asselberghs , A. Persoons , Angew. Chem. Int. Ed. 1999, 38, 366; 10.1002/(SICI)1521-3773(19990201)38:3<366::AID-ANIE366>3.0.CO;2-D 29711640

[7b] F. Paul , K. Costuas , I. Ledoux , S. Deveau , J. Zyss , J.-F. Halet , C. Lapinte , Organometallics 2002, 21, 5229;

[7c] C. Sporer , I. Ratera , D. Ruiz-Molina , Y. Zhao , J. Vidal-Gancedo , K. Wurst , P. Jaitner , K. Clays , A. Persoons , C. Rovira , J. Veciana , Angew. Chem. Int. Ed. 2004, 43, 5266; 10.1002/anie.200454150 15455435

[7d] P. Kaur , M. Kaur , G. Depotter , S. Van Cleuvenbergen , I. Asselberghs , K. Clays , K. Singh , J. Mater. Chem. 2012, 22, 10597;

[7e] S. Di Bella , I. P. Oliveri , A. Colombo , C. Dragonetti , S. Righetto , D. Roberto , Dalton Trans. 2012, 41, 7013; 22547326 10.1039/c2dt30702b

[7f] E. Cariati , X. Liu , Y. Geng , A. Forni , E. Lucenti , S. Righetto , S. Decurtins , S.-X. Liu , Phys. Chem. Chem. Phys. 2017, 19, 22573; 28809980 10.1039/c7cp04687a

[7g] I. Asselberghs , Y. Zhao , K. Clays , A. Persoons , A. Comito , Y. Rubin , Chem. Phys. Lett. 2002, 364, 279.

[8a] V. Guerchais , L. Ordronneau , H. Le Bozec , Coord. Chem. Rev. 2010, 254, 2533;

[8b] K. A. Green , M. P. Cifuentes , M. Samoc , M. G. Humphrey , Coord. Chem. Rev. 2011, 255, 2530;

[8c] F. Castet , V. Rodriguez , J.-L. Pozzo , L. Ducasse , A. Plaquet , B. Champagne , Acc. Chem. Res. 2013, 46, 2656; 23865890 10.1021/ar4000955

[8d] J. Boixel , V. Guerchais , H. Le Bozec , D. Jacquemin , A. Amar , A. Boucekkine , A. Colombo , C. Dragonetti , D. Marinotto , D. Roberto , S. Righetto , R. De Angelis , J. Am. Chem. Soc. 2014, 136, 5367; 24635126 10.1021/ja4131615

[8e] P. Beaujean , F. Bondu , A. Plaquet , J. Garcia-Amorós , J. Cusido , F. M. Raymo , F. Castet , V. Rodriquez , B. Champagne , J. Am. Chem. Soc. 2016, 138, 5052. 26996994 10.1021/jacs.5b13243

[9a] I. Asselberghs , K. Clays , A. Persoons , A. M. McDonagh , M. D. Ward , J. A. McCleverty , Chem. Phys. Lett. 2003, 368, 408;

[9b] L. Boubekeur-Lecaque , B. J. Coe , K. Clays , S. Foerier , T. Verbiest , I. Asselberghs , J. Am. Chem. Soc. 2008, 130, 3286; 18298121 10.1021/ja711170q

[9c] C. Karthika , S. R. Sarath Kumar , L. Kathuria , P. K. Das , A. G. Samuelson , Phys. Chem. Chem. Phys. 2019, 21, 11079. 31093630 10.1039/c9cp00946a

[10a] M. Sadakane , E. Steckhan , Chem. Rev. 1998, 98, 219. 11851504 10.1021/cr960403a

[11a] A. Al-Yasari , N. Van Steerteghem , H. El Moll , K. Clays , J. Fielden , Dalton Trans. 2016, 45, 2818; 26815652 10.1039/c6dt00115g

[11b] A. Al-Yasari , N. Van Steerteghem , H. Kearns , H. El Moll , K. Faulds , J. A. Wright , B. S. Brunschwig , K. Clays , J. Fielden , Inorg. Chem. 2017, 56, 10181; 28809116 10.1021/acs.inorgchem.7b00708

[11c] A. Al-Yasari , P. Spence , H. El Moll , N. Van Steerteghem , P. N. Horton , B. S. Brunschwig , K. Clays , J. Fielden , Dalton Trans. 2018, 47, 10415; 29947391 10.1039/c8dt01491d

[11d] E. Rtibi , M. Abderrabba , S. Ayadi , B. Champagne , Inorg. Chem. 2019, 58, 11210. 31390191 10.1021/acs.inorgchem.9b01857

[12] F. J. Carver , C. A. Hunter , D. J. Livingstone , J. F. McCabe , E. M. Seward , Chem. Eur. J. 2002, 8, 2847. 10.1002/1521-3765(20020703)8:13<2847::aid-chem2847>3.0.co;2-m 12489213

[13a] Y. Wei , B. Xu , C. L. Barnes , Z. Peng , J. Am. Chem. Soc. 2001, 123, 4083; 11457161 10.1021/ja004033q

[13b] I. Bar-Nahum , K. V. Narasimhulu , L. Weiner , R. Neumann , Inorg. Chem. 2005, 44, 4900. 15998013 10.1021/ic050473c

[14] Deposition number 2214337 contains the supplementary crystallographic data for this paper. These data are provided free of charge by the joint Cambridge Crystallographic Data Centre and Fachinformationszentrum Karlsruhe Access Structures service.

[15a] J. B. Strong , G. P. A. Yap , R. Ostrander , L. M. Liable-Sands , A. L. Rheingold , R. Thouvenot , P. Gouzerh , E. A. Maatta , J. Am. Chem. Soc. 2000, 122, 639;

[15b] B. Xu , Y. Wei , C. L. Barnes , Z. Peng , Angew. Chem. Int. Ed. 2001, 40, 2290; 11433497

[16] A. Al-Yasari , H. El Moll , R. Purdy , Kevin B. Vincent , P. Spence , J.-P. Malval , J. Fielden , Phys. Chem. Chem. Phys. 2021, 23, 11807. 33987634 10.1039/d0cp06610a

[17] A. Al-Yasari, Ph.D Thesis, University of East Anglia, **2016**.

[18] Previously, a maximum of 3 complete on/off cycles have been demonstrated by electrochemistry or redox switching (ref 7f), and 4 complete cycles by photo-switching (ref 8d) albeit with steady deterioration of the on-state signal.

