# Supplementary material for: Electrochemically‐Switched 2nd Order Non‐Linear Optical Response in an Arylimido‐Polyoxometalate with High Contrast and Cyclability
Source: Angew Chem Int Ed Engl. 2022 Dec 27;62(5):e202215537. doi: 10.1002/anie.202215537 (PMC10107823; doi:10.1002/anie.202215537)
Supplement: Supplementary file 1 — Supporting Information [file ANIE-62-0-s003.pdf]

Angewandte Chemie
Eine Zeitschrift der Gesellschaft Deutscher Chemiker

Supporting Information

Electrochemically-Switched 2nd Order Non-Linear Optical Response in an Arylimido-Polyoxometalate with High Contrast and Cyclability

B. R. Hood, Y. de Coene, A. V. Torre Do Vale Froes, C. F. Jones, P. Beaujean, V. Liégeois, F. MacMillan, B. Champagne*, K. Clays*, J. Fielden**

Supplementary information for:

Electrochemically-Switched Second Order Non-Linear Optical Response in an Imido-Polyoxometalate Derivative with High Contrast and Cyclablity

Bethany R. Hood,Yovan de Coene, Afonso V. Torre Do Vale Froes, Claire F. Jones, Pierre Beaujean, Vincent Liégeois, Fraser MacMillan, Benoît Champagne, Koen Clays and John Fielden

General

Materials and Procedures. Acetonitrile (MeCN) for synthesis and electrochemistry was freshly distilled over CaH_2 under a nitrogen atomsophere.[1] Dry dimethyl sulfoxide (DMSO) was purchased from Sigma Aldrich (SureSeal) and Acros Organics (Acro Seal). Acetonitirile for HRS switching studies was purchased dry from Sigma Aldrich in SureSeal bottles and used as received. All other reagents and solvents were obtained as ACS grade from Sigma Aldrich, Alfa Aesar, Fisher Scientific, Fluorochem, Acros Organics, or Apollo Scientific and used as supplied. Deuterated solvents were obtained from Eurisotop, Cambridge Isotope Laboratories, or Acros Organics and used as supplied. Tetrabutylammonium hexamolybdate[2] was synthesized according to previously published methods, 4-dimethylamino-2,6-diisopropylaniline and its derivatization with hexamolybdate were achieved following adapted synthetic procedures.[3,4] Spectroscopic data obtained for **P1** to **P5** matched that previously published, so these were not characterized further. The hexamolybdate derivatization chemistry was performed under an atmosphere of dry argon using standard Schlenk techniques. Unless otherwise stated, preparation of organic precursors was carried out in air.

General Physical Measurements. FT-IR spectra were measured using a Bruker FT-IR XSA spectrometer. ^{1}H- and ^{13}C-NMR spectra were acquired using Bruker AC 300 (300 MHz) and Bruker Ascend 500 (500 MHz) spectrometers and all shifts are quoted with respect to TMS using the solvent signals as secondary standard (s = singlet, d = doublet, t = triplet, q = quartet, sex = sextet, hept = heptet, dt = doublet of triplets, m = multiplet). Quaternary carbon signals were not observed for these compounds even after 1064 scans of saturated d_6-DMSO solutions, which gave strong signal for all other ^{13}C resonances. Elemental analyses and accurate mass spectrometry were outsourced to the University of Manchester, and the John Innes Centre (Norwich) respectively. UV–Vis spectra were obtained by using an Agilent Cary 60 UV-Vis spectrophotometer.

Synthetic Methods

Our synthetic route to compound **1** is shown in Scheme S1.

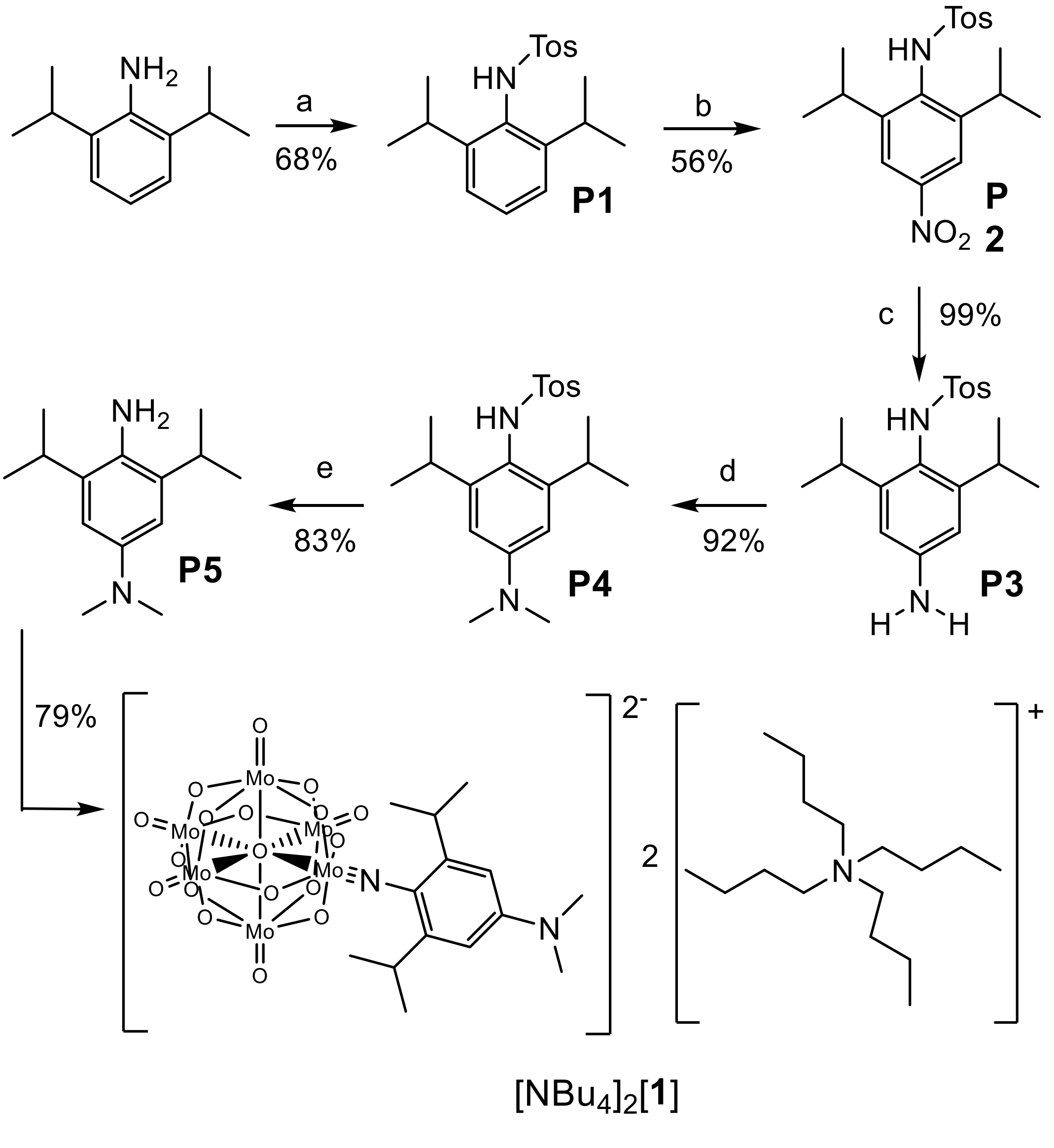

Scheme S1 Synthetic route to componund $[NBu_4]_2$[**1**]. Reagents and conditions: (a) TsCl, pyridine, reflux, 4 h. (b) HNO_3, $NaNO_2$, AcOH, H_2O, reflux, 12 h. (c) $SnCl_2{\cdot}2H_2O$, EtOH, reflux, 23 h. (d) H_2CO, HCl, $NaBH_3$, THF/H_2O, 15 °C, 2 min. (e) 95% H_2SO_4, 40 °C, 16 h. (f) $[NBu_4]_2[Mo_6O_{19}]$, DCC, DMSO, 70 °C, 10 h.

Preparation of N-tosyl-2,6-diisopropylaniline (P1)

The preparation of compound **P1** was carried out under an argon atmosphere using standard Schlenk techniques. Toluene sulfonyl chloride (10.124 g, 47.5 mmol) and 2,6-diisopropylaniline (9 ml, 8.46 g, 47.7 mmol) dissolved in 20 ml dry pyridine to give a yellow solution which was refluxed for 4 hours. The resulting orange solution was poured into 60 ml of 2M hydrochloric acid to give an orange solid which was collected by filtration and purified by recrystallisation in hot ethanol to give compound **P1** as colourless crystals (10.781 g, 32.5 mmol) in a 68% yield. ^{1}H-NMR (500 MHz, $CDCl_3$) δ 7.59 (2H, d, *J* = 8.3 Hz), 7.25-7.20 (3H, m, 5.0 Hz), 7.09 (2H, d, *J* = 7.7 Hz), 6.13 (1H, s), 3.12 (2H, hept, *J* = 6.8 Hz), 2.39 (3H, s), 0.99 (12H, d, *J* = 6.9 Hz).

Preparation of N-tosyl-4-nitro-2,6-diisopropylaniline (P2)

To a mixture of compound **P1** (3.505 g, 10.6 mmol), 70 ml of water, and 15 ml nitric acid, 70 ml of glacial acetic acid was added followed by sodium nitrite (1.115 g, 16.2 mmol). The mixture was refluxed for 12 hours and then, once cooled, poured into 200 ml of water to give a pale yellow solution with a pale precipitate. Over the course of 2 days, pale yellow crystals formed which were then collected by filtration, washed with water, and dried in vacuo to give compound **P2** (2.242 g, 5.96 mmol) in a 56% yield. ^{1}H-NMR (500 MHz, $CDCl_3$) δ 7.97 (2H, s), 7.59 (2H, d, *J* = 7.4 Hz), 7.28 (2H, d, *J* = 8.0 Hz), 6.19 (1H, s), 3.18 (2H, hept, *J* = 6.8 Hz), 2.43 (3H, s), 1.06 (12H, d, *J* = 6.8 Hz).

Preparation of N-tosyl-4-amino-2,6-diisopropylaniline (P3)

Preparation of **P3** was carried out under an argon atmosphere using standard Schlenk techniques. **P2** (0.101 g, 0.269 mmol) and tin(II) chloride dihydrate (0.200 g, 0.105 mmol) were dissolved in 6 ml degassed ethanol with heating. The resulting yellow solution was refluxed for 23 hours and then poured over ice to give a white precipitate. The resulting mixture was made basic with sodium bicarbonate and then extracted with ethyl acetate. The organic layer was dried over magnesium sulfate and then the solvent was removed in vacuo to give compound **P3** as a pale yellow solid (0.092 g, 0.266 mmol) in a 99% yield. ^{1}H-NMR (500 MHz, $CDCl_3$) δ 7.60 (2H, d, *J* = 8.3 Hz), 7.23 (2H, d, *J* = 7.2 Hz), 6.39 (2H, s), 6.12 (1H, s), 3.65 (2H, s), 3.03 (2H, hept, *J* = 6.6 Hz), 2.40 (3H, s), 0.95 (12H, d, *J* = 6.8 Hz).

Preparation of N-tosyl-4-dimethylamino-2,6-diisopropylaniline (P4)

A slurry of compound **P3** (0.970 g, 2.80 mmol) and sodium borohydride (0.817 g, 21.6 mmol) in 25 ml of tetrahydrofuran was slowly added to a solution of 40% aqueous formaldehyde (1.4 ml, 15.2 mmol) in 3 M hydrochloric acid (3 ml, 0.009 mmol) whilst keeping the temperature below 15°C using an ice bath. When the addition was complete the mixture was made basic using sodium hydroxide and then the organic layer was decanted. The remaining slurry was extracted twice with ether and then the organic fractions were combined and dried over magnesium sulfate. The solvent was removed in vacuo to give compound **P4** as an off white solid (0.966 g, 2.58 mmol) in a 92% yield. ^{1}H-NMR (500 MHz, $CDCl_3$) δ 7.62 (2H, d, *J* = 8.3 Hz), 7.24 (2H, d, *J* = 8.0 Hz), 6.42 (2H, s), 5.79 (1H, s), 3.07 (2H, sept, *J* = 6.3 Hz), 2.95 (6H, s), 2.40 (3H, s), 0.99 (12H, d, *J* = 6.8 Hz).

Preparation of 4-dimethylamino-2,6-diisopropylaniline (P5)

Compound **P4** (0.929 g, 2.48 mmol) was dissolved in 15 ml of 95% sulfuric acid and the resulting brown solution was left to stir at 40°C overnight. The resulting dark brown solution was then poured over ice and made basic with sodium hydroxide before extracting into DCM. The orange organic layer was dried over magnesium sulfate before the solvent was removed in vacuo to give compound **P5** as a purple oil (0.451 g, 2.05 mmol) in an 83% yield. ^{1}H-NMR (500 MHz, $CDCl_3$) δ 6.53 (2H, s), 3.32 (2H, s), 2.90 (2H, hept, *J* = 6.7 Hz), 2.77 (6H, d, *J* = 7.1 Hz), 1.20 (12H, d, *J* = 6.8 Hz). FTIR: 2957 (s), 2868 (m), 2828 (w), 2782 (m), 1602 (s), 1481 (s), 1437 (sh), 1382 (w), 1353 (m), 1256 (m), 1216 (m), 1096 (m), 983 (s), 927 (w), 842 (m), 755 (m). UV-vis (MeCN) λ, nm (ε, M^{-1} cm^{-1}): 206 (26.7 $\times 10^3$); 256 (11.7 $\times 10^3$); 318 (3.04 $\times 10^3$).

Preparation of [$(C_4H_9)_4N]_2[Mo_6O_{18}N_2C_{14}H_{22}$] ([$NBu_4]_2$[1]])

To compound [$NBu_4]_2[Mo_6O_{19}$] (0.999 g, 0.732 mmol) and DCC (0.170 g, 0.824 mmol), 10 ml of dry DMSO and **P5** (0.153 g, 0.694 mmol) were added. The resulting dark green solution was heated at 70ºC for 10 hours. When cool, the red solution was filtered to remove the pale precipitate and then poured into 40 ml of ethanol and 230 ml of diethyl ether. The resulting dark precipitate was collected by filtration, washed with ethanol and ether and then dried in vacuo to give crude compound **8** as a brown solid (0.911 g, 0.581 mmol) with a 79% yield. Further purification of 0.571 mg of sample was achieved by crystallisation from hot acetonitrile to give pure compound **8** (0.322 g, 0.206 mmol) in an overall 45% yield. ^{1}H-NMR (500 MHz, CD_3CN) δ 6.39 (s, 2H), 3.82 (hept, *J* = 6.8 Hz, 2H), 3.14 – 3.05 (m, 22H), 1.66 – 1.55 (m, 16H), 1.36 (hex, *J* = 7.4 Hz, 16H), 1.29 (d, *J* = 6.8 Hz, 12H), 0.97 (t, *J* = 7.4 Hz, 24H). ^{13}C-NMR (126 MHz, CD_3CN) δ 152.25, 106.08, 59.52, 40.51, 30.02, 24.54, 24.36, 20.53, 14.01. Anal (Calcd) % for $C_{46}H_{94}N_4O_{18}Mo_6$: C, 35.26 (35.11); H, 6.05 (5.98); N, 3.58 (3.58). HRMS (ESI, MeCN) = calcd for $C_{14}H_{22}N_2Mo_6O_{18}^{2-}$ 540.7617, found 540.7609. FTIR: 2959 (m), 2932 (sh), 2872 (m), 1590 (m), 1480 (m), 1462 (sh), 1372 (m), 1313 (m), 1233 (w), 1170 (m), 1125 (vw), 1108 (vw), 1065 (vw), 1029 (vw), 997 (w), 970 (m), 940 (s), 880 (w), 772 (s). UV-vis (MeCN) λ, nm (ε, M^{-1} cm^{-1}): 206 (44.3×10^3); 258 (28.1×10^3); 431 (30.7×10^3).

X-ray Crystallographic Details

Sample Growth, Data Collection and Refinement. Crystals of [$NBu_4]_2$[**1**]·Me_2CO were grown by diffusion of diethyl ether into an acetone solution. Data were collected on a Rigaku XtalLab Synergy S diffractometer using a Photon-Jet Cu microfocus source and Hypix hybrid photon counting detector. Data reduction, cell refinement and absorption correction were carried out using Rigaku CrysAlisPro,[5] and the structure was solved with SHELXT[6] in Olex2 V1.5.[7] Refinement was achieved by full-matrix least-squares on all F_0^2 data using SHELXL (v. 2018-3),[8] also in Olex 2 V1.5. The asymmetric unit contains the complete molecular anion, both tetrabutylammonium cations and a DMSO solvent molecule (see Figure S1) originating from the original DMSO-based synthesis. Disorder on two tetrabutylammonium side chains was modelled requiring application of restraints to bond distances and thermal parameters.

Table S1. Crystallographic Data and Refinement Details for [$NBu_4]_2$[**1**]·Me_2CO

	**1**
Formula	$C_{49}H_{100}Mo_6N_4O_{19}$
M	1624.96
crystal system	triclinic
space group	*P*-1
a/Å	12.2689(1)
b/Å	24.3437(2)
c/Å	25.2376(2)
α/deg	115.515(1)
β/deg	102.332(1)
γ/deg	94.348(1)
V/Å^3	6524.13(1)
Z	4
Radiation	Mo Kα (*λ* = 0.71073 Å)
T/K	100.01(1)

μ/mm^{-1}	1.185
Cryst. size/mm	0.19 × 0.137 × 0.043
Cryst. description	Orange plate
No. reflns collected	109378
No. of indep. reflns (R_{int})	33135 [R(int) = 0.0358]
$2\theta_{full}$/deg (completeness)<br>$2\theta_{max}$/deg (completeness)	50.484 (99.9%)<br>61.79 (80.4%)
Reflections with $I > 2\sigma(I)$	26595
Goodness-of-fit on F^2	1.012
final R_1, wR_2 $[I > 2\sigma(I)]^a$	R1 = 0.0335, wR2 = 0.0726
(all data)	R1 = 0.0486, wR2 = 0.0771
Peak and hole/e Å^{-3}	1.10 and -0.65

Structures and Selected Bond Lengths. A representation of the asymmetric unit of $[NBu_4]_2$[**1**]·Me_2CO is shown in Figure S1. Selected bond lengths and angles for the anion [**1**]$^{2-}$ are collected in Tables S2 and S3, together with those of the isopropyl-free analogue [**1H**]$^{2-}$ (ref 11b, main paper). Both structures show the typical imido-Lindqvist pattern of a shortened bond length from the imido-Mo (Mo^{im}) to the central oxygen (O^c), lengthened equatorial bond lengths from Mo^{im} to the oxygens bridging to the belt Mo positions (Mo^b), and a lengthened axial bond length from the *trans*-Mo (Mo^t) to O^c (see references 13a and 14, main paper). There is no pattern in the terminal Mo=O distances which are typically in the range of 1.65 to 1.70 Å. The main difference between the structures is the slightly longer and much more linear Mo-N-C bond angle in [**1**]$^{2-}$ forced by the isopropyl groups. Bond distances within the imido-aryl ligands suggest a less quinoidal structure (i.e. weakened donor acceptor communication, borne out by electrochemical measurements) in [**1**]$^{2-}$, but differences are close to being within experimental errors.

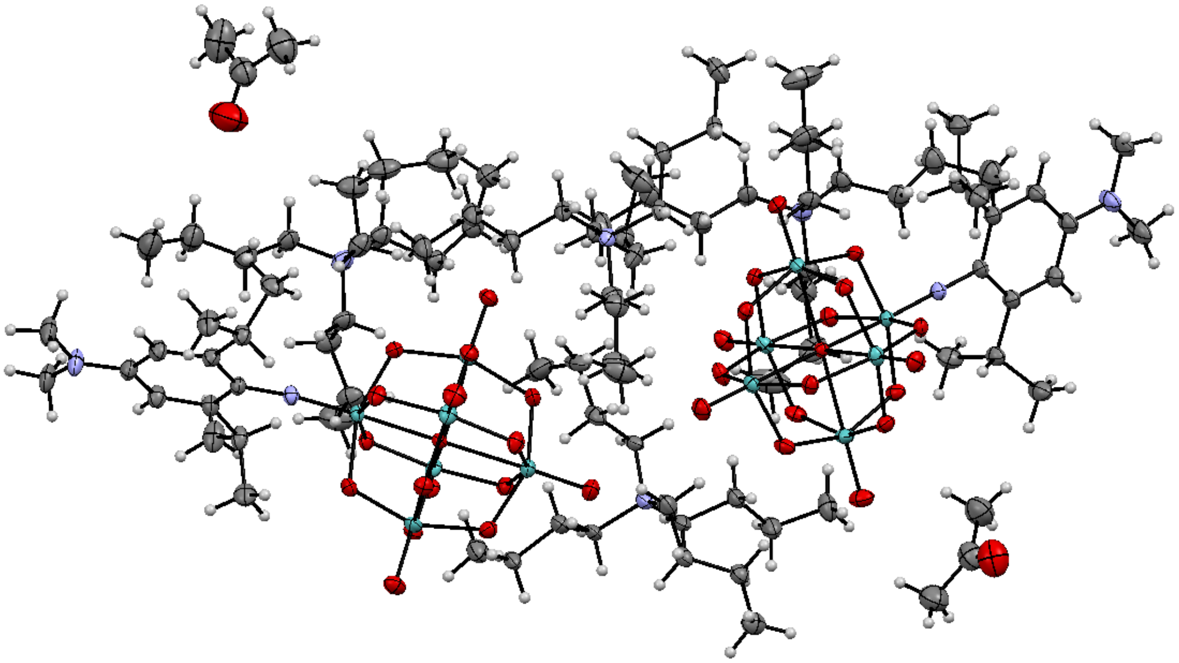

Figure S1 ORTEP representation of the asymmetric unit in $[NBu_4]_2$[**1**]·Me_2SO. Thermal ellipsoids are at the 30% probability level and disordered parts are omitted for clarity. Colour scheme: Mo is green; O, red; C, gray; N, blue; S, yellow; H atoms are represented by white spheres of arbitrary radii.

Table S2 Selected bond lengths (Å) and angles (°) of the {Mo_6} units in anions [**1**]$^{2-}$ and [**1H**]$^{2-}$. Mo^{im} – imido bearing Mo; Mo^b belt Mo; Mo^t, *trans*-Mo; O^c, central O; O^b, bridging O.

	Mo^{im}-N	Mo^{im}-O^c	Mo^{im}-O^b (average)	Mo^t-O^c	Mo^t-O^b (average)	O^c-Mo^{im}-N	Mo^{im}-N-C
[**1**]$^{2-}$ [*a*]	1.741(1)	2.192(2)	1.957(2)	2.358(3)	1.921(2)	177.0(1)	175.0(2)
[**1H**]$^{2-}$ [*b*]	1.737(3)	2.210(2)	1.955(3)	2.350(2)	1.945(3)	173.7(1)	162.6(3)

[*a*]Average of two crystallographically independent, chemically identical anions. [*b*]Previously published in ref 11a (main paper).

Table S3 Bond lengths in the arylimido groups of anions [**1**]$^{2-}$ and [**1H**]$^{2-}$, showing the quinoidal structure induced by connection to the electron withdrawing POM.

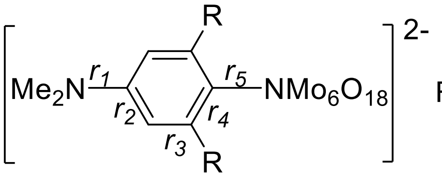

R = ^{i}Pr (**1**), H (**1H**),

	***r*1**	***r*2**[*a*]	***r*3**[*a*]	***r*4**[*a*]	***r*5**
[**1**]$^{2-}$	1.363(4)	1.414(4)	1.377(4)	1.416(4)	1.368(3)
[**1H**]$^{2-}$ [*b*]	1.375(5)	1.421(6)	1.355(6)	1.407(5)	1.383(5)

[*a*]Average of two chemically identical, crystallographically independent C-C bonds. [*b*]Previously published in ref 11a (main paper).

Electrochemistry

Cyclic Voltammetry. All measurements were carried out using Autolab PGStat 302 potentiostat/galvanostat. A single-compartment or a conventional three-electrode cell was used with an Ag wire pseudo reference electrode, glassy carbon working electrode and Pt wire auxiliary electrode. Acetonitrile was freshly distilled (from CaH_2), $[N(C_4H_9\text{-}n)_4]PF_6$, as supplied from Fluka, and $[N(C_4H_9\text{-}n)_4]BF_4$,[9] were used as the supporting electrolyte. Solutions containing ca. 10^{-3} M analyte (0.1 M electrolyte) were degassed by purging with argon and blanketed with a continuous flow of argon throughout the experiments. $E_{1/2}$ values were calculated from $(E_{pa} + E_{pc})/2$ at a scan rate of 100 mV s^{-1} and referenced to Fc/Fc^+.

Bulk Electrolysis. Bulk electrolysis was performed in a three compartment cell with counter and reference electrodes segregated from the working compartment by sintered glass disks to minimize potential drift and prevent re-oxidation of analyte. A platinum gauze working electrode was used, with Ag wire reference and Pt counter and vigorous stirring was applied. An additional glassy carbon working electrode was used to measure cyclic voltammograms before and after reduction. Acetonitrile was freshly distilled (from CaH_2) and $[N(C_4H_9\text{-}n)_4]BF_4$,[9] was used as the supporting electrolyte. Solutions containing ca. 0.8 mM analyte (0.1 M electrolyte) were degassed by purging with argon and blanketed with a continuous flow of argon throughout the experiments. Reduction of the bulk sample was achieved by application of a potential of -0.7 V vs $Ag^{0/+}$ to the rigorously stirring solution for a total of 22 minutes. After this time the current had plateaued at a current of near 0 A showing no further reduction was taking place.

Spectroelectrochemistry. All measurements were performed using a Spectroelectrochemistry Reading RT OTTLE cell,[10] with an Agilent Cary 60 UV-vis spectrophotometer and Autolab μ-III potentiostat. The analyte concentration was *ca.* 8×10^{-3} M in 0.3 M NBu_4BF_4 in dry acetonitrile. A reductive potential of between -0.8 and -0.5 V vs Ag was applied for approximately two minutes, varied to account for potential drift determined by a cyclic voltammogram taken immediately prior. UV-vis spectra were recored at 15 second intervals. When no further changes were observed, a re-oxidising potential of -0.3 to 0 V vs Ag was applied for approximately two minutes until the continutes UV-vis monitoring showed no further changes.

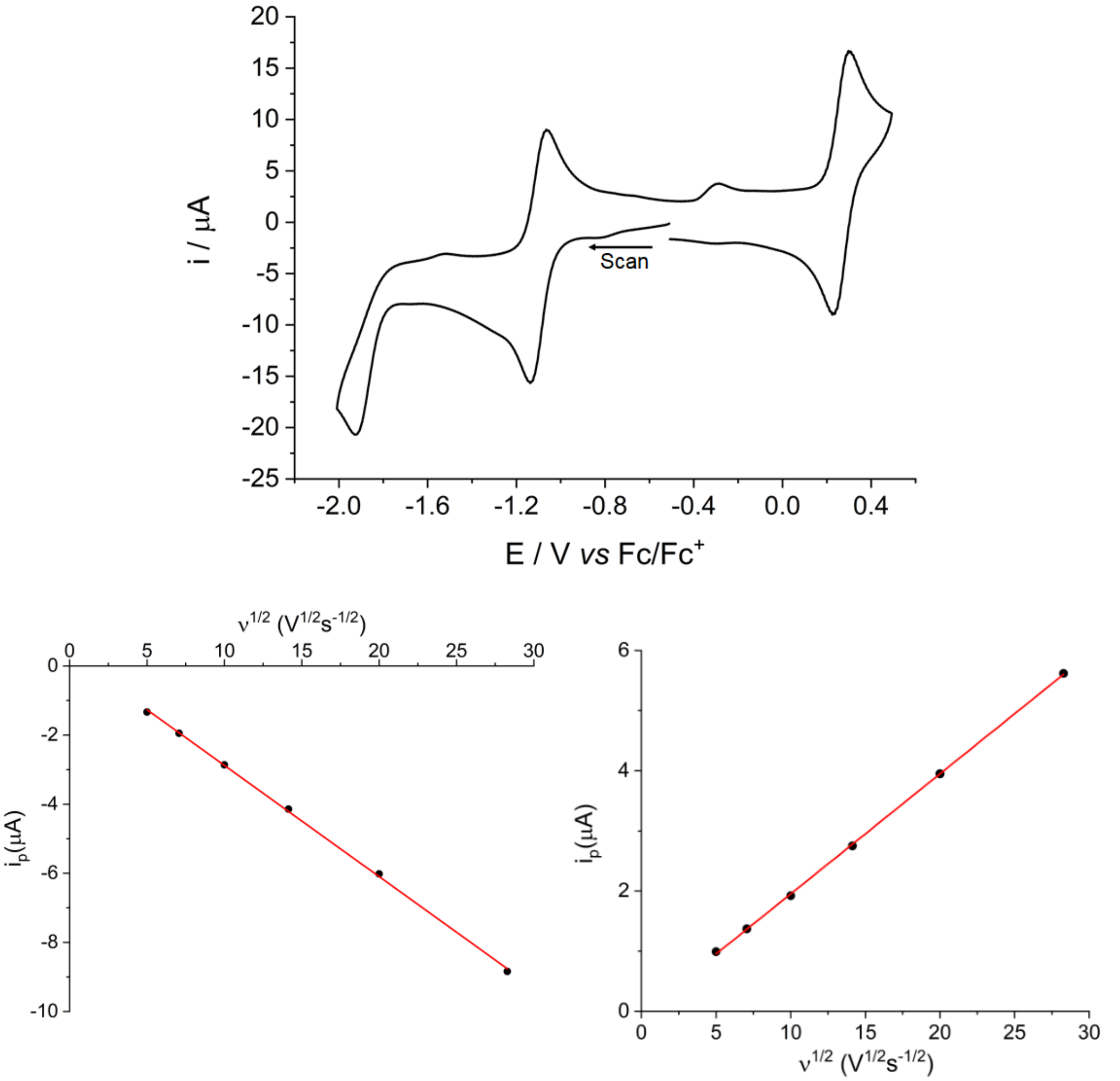

Figure S2 Top: Full range cyclic voltammogram of $[NBu_4]_2$[**1**], showing the reversible POM reduction peak at -1V $^+$, destructive POM reduction peak at *ca.* -1.9 V, and amine oxidation peak at +0.30 V (all *vs* Fc/Fc^+). Scan rate 100 mV s^{-1}, GC working electrode, 0.1 M $[NBu_4][BF_4]$ in acetonitrile as electrolyte. **Bottom:** Peak current *vs* square root of scan rate for the first POM reduction (**left**) and amine oxidation (**right**), indicating a freely diffusing species.

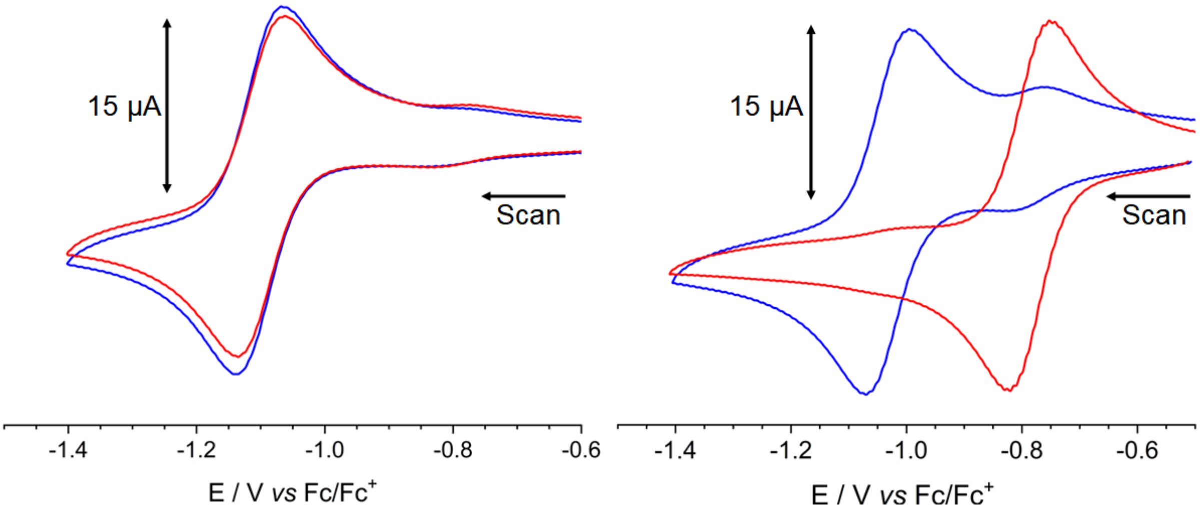

Figure S3 Cyclic voltammograms of $[NBu_4]_2$[**1**] (**left**) and $[NBu_4]_2$[**1H**] (right) before (blue) and after bulk electrolysis for 22 min at -0.7 V vs Ag/Ag^+, showing minimal production of $[Mo_6O_{19}]^{2-}$ or loss of $[\mathbf{1}]^{2-/3-}$. To aid comparison, the baseline current of the post-BE scans at the start potential have been shifted to the same value as the initial scan.

Quantum chemistry calculations

Geometry optimizations. Full geometry optimizations were performed at the density functional theory (DFT) level using the ωB97X-D exchange-correlation functional (XCF).[11] This range-separated hybrid functional combines a modified B97 exchange functional[12] for short-range interactions (from 78 to 0%) with HF exchange for long-range interactions (from 22 to 100%), using the default range-separating parameter, ω = 0.2 $Bohr^{-1}$. Correlation is described by the B97 correlation functional,[12] and empirical atom-atom London dispersion corrections are included as well. For the C, H, N, and O atoms, the atomic basis set consists of 6-311G(d,p)[13] while LANL2TZ[14] is used for the Mo atoms. TIGHT convergence thresholds on the residual forces on the atoms (1.5 x 10^{-5} Hartree/Bohr or Hartree/radian) were applied. The reliability of the ωB97X-D/6-311G(d,p)/LANL2TZ method for the geometry optimization of POM derivatives was demonstrated in comparison with other XC functionals in a previous work.[15] To describe the solvent effects (acetonitrile), geometry optimizations were performed in solution using the integral equation formalism (IEF) of the polarizable continuum model (PCM) (IEF-PCM), which represents the solvent by a dielectric continuum characterized by its dielectric permittivity (ε_0 = 35.688 for acetonitrile).[16]

Properties of the excited states. Using the optimized geometries, the time-dependent density functional theory (TD-DFT) method[17] with the same XCF, basis set, and IEF-PCM scheme was used to characterize the (lowest-energy) excited states, considering their vertical excitation energies, $\Delta E_{ge} = E_e - E_g$ (from the ground state g to the excited state e), the corresponding transition dipole moment, μ_{ge}, which is related to the oscillator strength, $f_{ge} = \frac{2}{3}\,\Delta E_{ge}\,\mu_{ge}^2$, and the difference of dipole moment between states g and e, $\Delta\mu_{ge} = \mu_e - \mu_g$. The excited state dipole moments were calculated by using the nonequilibrium IEF-PCM solvation approach (ε_∞ = 1.807 for acetonitrile). Nonequilibrium solvation TDDFT calculations also provided the

difference of electron density between the excited and the ground states, $\Delta\rho(\vec{r}) = \rho_e(\vec{r}) - \rho_g(\vec{r})$. Following Le Bahers *et al*,[18] the barycenters of the positive $[\Delta\rho^+(\vec{r})]$ and negative $[\Delta\rho^-(\vec{r})]$ electron density variations were calculated and the distance between them defines the charge-transfer distance (d_{CT}) while the integration over the whole space of $\Delta\rho^+(\vec{r})$ [or $\Delta\rho^-(\vec{r})$] gives the amount of charge transferred (q_{CT}). The product of these two quantities, $q_{CT} \times d_{CT}$, gives $\Delta\mu_{ge}$. For the oxidized form (closed-shell species), the 120 lowest excitation energies (and oscillator strengths) were calculated whereas 240 were obtained for the reduced form (open-shell species).

First hyperpolarizabilities. Using again the same optimized geometries, the SHG β tensor components were evaluated by employing the quadratic response TD-DFT method[19,20] with the same basis set, XCF, and solvation model as for the excited states calculations. Both static and dynamic (incident wavelength of 1064 nm) responses were calculated. Computing β of large compounds, including those having donor and/or acceptor substituents, is a challenge for TD-DFT because of the intrinsic nonlocal nature of the response and the approximate XCFs (potential and kernel).[21] However, ωB97X-D is a reliable XCF for calculating the β tensors owing to its substantial amount of long-range HF exchange, as demonstrated in previous investigations where the performance of DFT XCFs was assessed with respect to benchmark wavefunction methods.[22-24] Ref. 15 has also evidenced that using local XCFs or XCFs with small amount of HF exchange leads to overestimated first hyperpolarizabilities. The unit sphere representation (USR) was adopted[25] to visualize the β tensors. First, the induced electric dipole moments

$$\vec{\mu}_{ind} = \overleftrightarrow{\beta} : \vec{E}^2(\theta, \phi) \quad (3)$$

are evaluated, where $\overleftrightarrow{\beta}$ is the first hyperpolarizability tensor and $\vec{E}(\theta, \phi)$ is a unit vector of electric field, of which the polarization is defined by the θ and ϕ angles (spherical coordinates). Then, the induced dipoles are plotted on a sphere centered on the molecule center of mass. This allows highlighting the directions along which the second-order polarizations are the strongest (i.e. the largest induced dipoles), its orientation (the acceptor-donor direction).

The method for β calculations accounts for the frequency dispersion of the β response but not for the fact that the UV/visible absorption band has a finite bandwidth. In these TD-DFT calculations the bandwidth is assumed to be zero. Far from resonance, this effect is negligible but when the SHG wavelength falls within the absorption band, such as for $[\mathbf{1}]^{2-}$ (Figure 3, main paper), the impact has to be taken into account. This appears also to be important when comparing the oxidized and reduced forms of **1** because for the latter, the lowest-energy absorption band is shifted towards the blue and the SHG wavelength is outside the absorption band. As a matter of fact, the two-state approximation (TSA) was enacted to provide β responses, more consistent with the measured values. First, the TD-DFT values ($\lambda = 1064\ nm$) were extrapolated by using the simple TSA with a zero bandwidth. Then, these static values are multiplied by a frequency-dependent term that accounts for the shape of the UV/visible absorption spectrum, following the inhomogeneous broadening single-mode model proposed by Campo *et al.*[26] That model, which implicitly contains information on the

distribution of the transition frequencies and takes into account the vibronic structure of the excited states, was already used in our previous studies.[27]

Computer codes. All DFT and TD-DFT calculations were performed using the Gaussian16 package.[28] Then, the molecular structures, electron densities, and USR pictures were generated using the DrawMol software.[29] The frequency dispersion factors were evaluated using a homemade code.

TD-DFT UV/visible absorption. The absorption spectrum of the oxidized form (Figure S4) presents an intense band peaking at 393 nm, originating from a single charge-transfer excitation (3.16 eV, f = 1.02) and a broad band having a maximum at ~250 nm, associated with many electronic transitions. The spectrum of the reduced form (Figure S4) is similar with a dominant transition at 369 nm (3.36 eV, f = 0.98) and a broad band around 240-260 nm, again associated with a huge number of electronic transitions. The 0.2 eV blue shift of the lowest-energy band is consistent with the 0.24 eV shift observed in the experimental spectrum (2.88 eV for the oxidized form versus 3.12 eV for the reduced one). The fact that the calculated spectrum is shifted to the blue with respect to experiment (3.16 eV versus 2.88 eV for the oxidized form and 3.36 eV versus 3.12 eV for the reduced form) was expected since the calculations report vertical excitation energies but do not account for the geometry relaxation of the excited state, neither for the vibronic structure. The presence of a higher-energy band close to 250 nm is also in agreement with experiment, though a closer match would have been achieved by including even more excited states in the TD-DFT calculations. The charge-transfer character of the dominant low-energy excitation is evidenced by the $\Delta\rho(\vec{r})$ plots (Figure S5). For the oxidized form, $\Delta\mu_{ge}$ = 9.6 D, oriented from the NMe_2 donor group towards the POM. In the case of the reduced form, the change of dipole moment is oriented in the same direction but it is slightly smaller (8.0 D), which is mostly due to a reduction of d_{CT} (2.8 Å versus 3.2 Å). This reduction is also visible in Figure S5 because there is an increase of density on the core of the POM for the oxidized form whereas it is negligible for the reduced one.

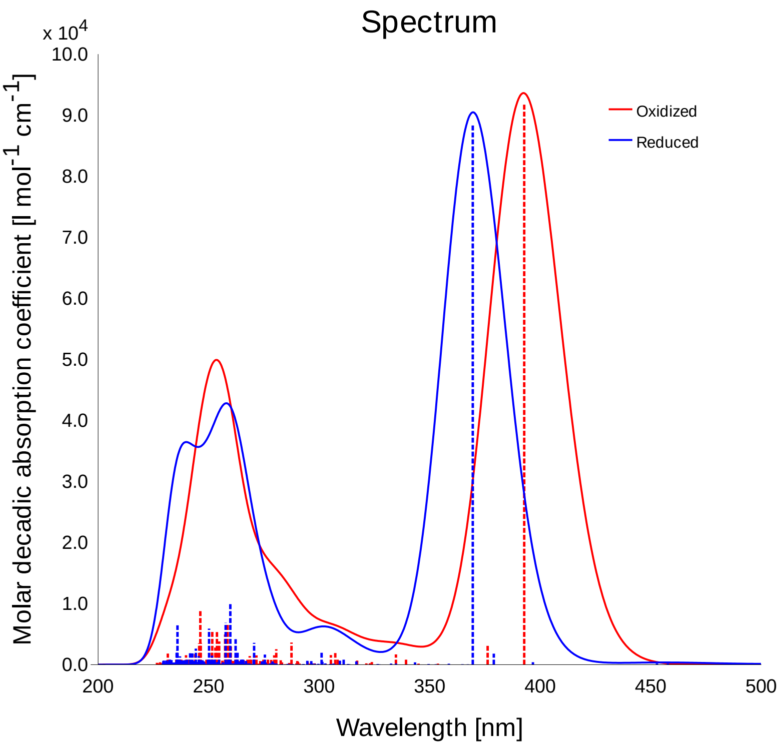

Figure S4. Simulated TD-DFT UV/visible absorption spectra of **1** in oxidized and reduced forms. The vertical bars represent the oscillator strengths of the different electronic transitions. The convoluted spectrum was obtained by associating to each transition a Gaussian function of half width at half maximum of 0.3 eV.

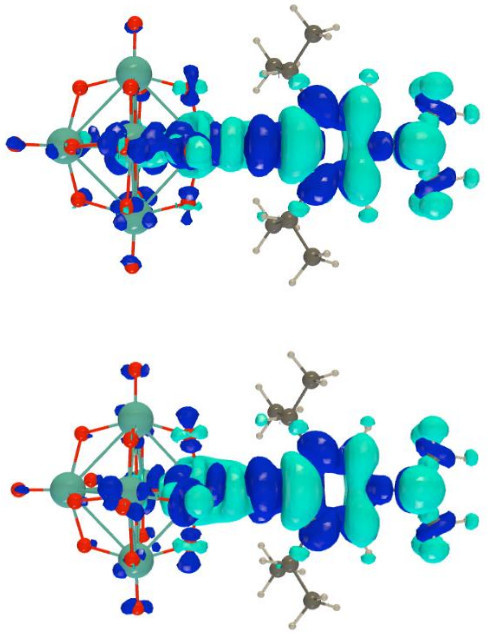

Figure S5. Excitation-induced electron density difference [$\Delta\rho(\vec{r})$] as calculated for the dominant low-energy excited state of the oxidized (top) and reduced (bottom) forms at the IEF-PCM/TD-DFT/ωB97X-D/6-311G*/LanL2TZ level of approximation [iso-value = 0.0006 a.u.; light/dark blue corresponds to negative/positive $\Delta\rho(\vec{r})$ so that the excitation-induced electron transfer goes from the light to the dark blue].

First hyperpolarizabilities

Table S4. Calculated and experimental β_{HRS} values for [**1**]$^{2-}$ and [**1**]$^{3-}$ and their ratios. See the text for the explanation of the two corrections to the calculated TD-DFT values.

10^{-30} esu (B convention)	β_{HRS} $[TD - DFT]$ $\lambda = 1064\,nm$	β_{HRS} $[TD - DFT + TSA]$ $\lambda = \infty$	β_{HRS} $[TD - DFT + TSA + inhomog.\, broad.\, single\ mode\ model]$ $\lambda = 1064\,nm$	$\beta_{HRS}\,[Exp.]$ $\lambda = 1064\,nm$
[**1**]$^{2-}$	73.9	29.1	149.0	127.9
[**1**]$^{3-}$	45.8	20.9	57.9	8.3
Ratio	1.6	1.4	2.6	15.4

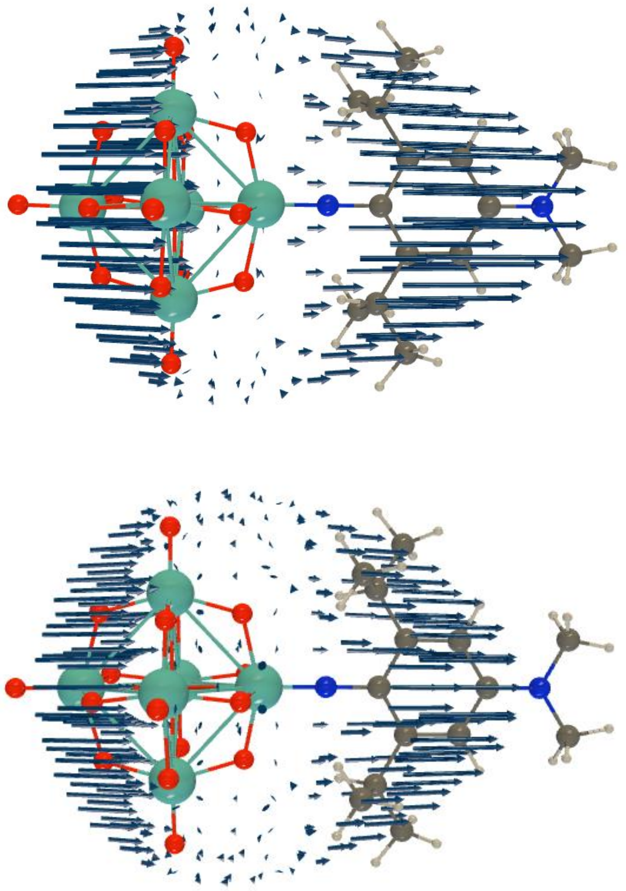

Figure S6. Unit sphere representation (USR) of the first hyperpolarizability tensor (λ = 1064 nm) of the oxidized (top) and reduced (left) species as calculated at the IEFPCM(solvent = acetonitrile) TDDFT/ωB97X-D/6-311G(d)/LanL2TZ level of approximation. Factor = 10^{-4} au of β/au of length. Using USR, the arrows (second-order field-induced induced dipoles) go from the acceptor to the donor groups.

EPR Spectroscopy

Methods and Instrumentation. Samples of $[Mo_6O_{19}]^{3-}$ and $[\mathbf{1}]^{3-}$ were prepared by bulk electrolysis of $[NBu_4]_2[Mo_6O_{19}]$ (0.02 mM) and $[NBu_4]_2[\mathbf{1}]$ (0.07 mM) in 0.1 M $[NBu_4][BF_4]$ electrolyte in acetonitrile. These were syringed into EPR tubes pre-purged with argon and then rapidly frozen in liquid nitrogen. These were syringed into standard 4mm quartz EPR tubes (Wilmad) pre-purged with argon and then rapidly frozen in liquid nitrogen. EPR spectra were obtained on a Bruker E560 EleXsys spectrometer equipped with a standard Bruker EPR cavity (ER4123 SHQE) and an Oxford instruments flow cryostat (ESR900) using liquid nitrogen (67 K) or liquid helium (10 K). Experimental parameters: microwave power, 0.1 mW (figure S7a) and 3.15 μW (figure S7b); field modulation frequency, 100 kHz; field modulation amplitude, 0.5 mT (figure S7a) and 0.8 mT (figure S7b). All spectral simulations were performed using the MATLAB-based EasySpin package[30]

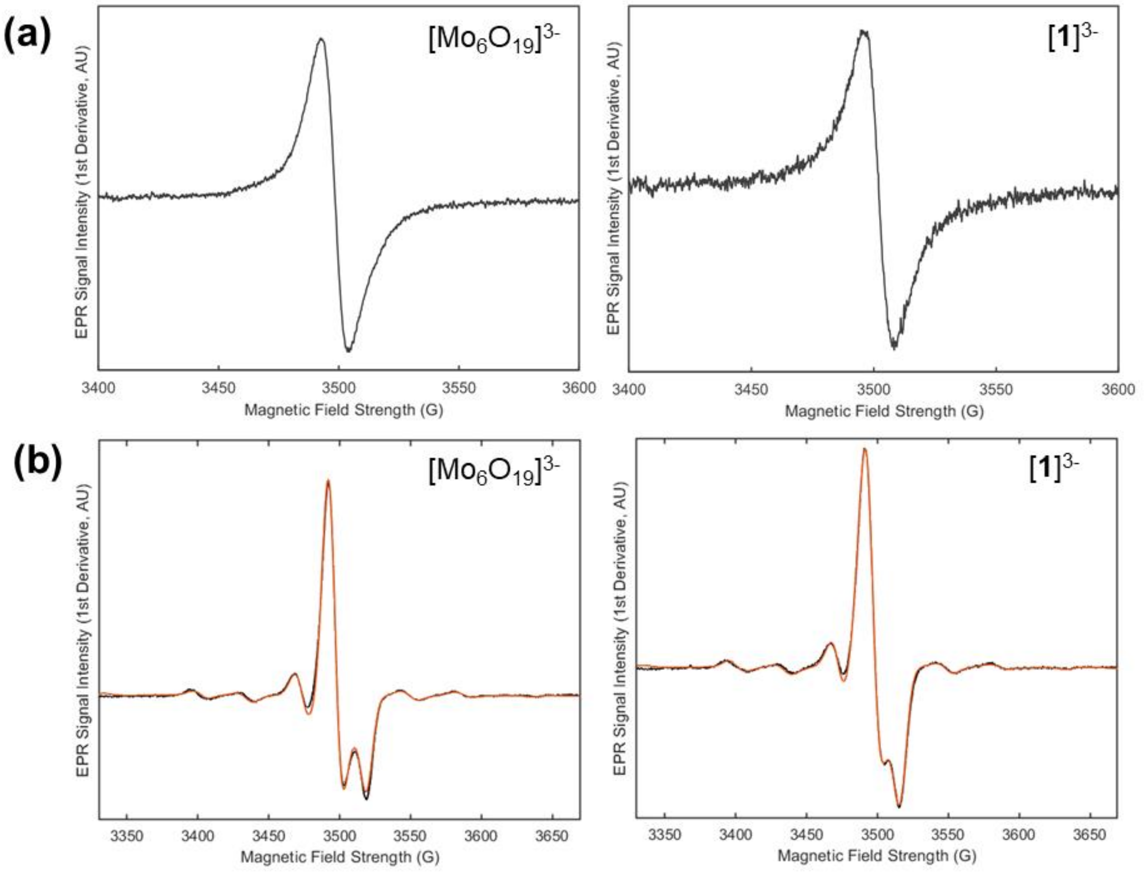

Figure S7 X-band EPR spectra of $[Mo_6O_{19}]^{3-}$ and arylimido derivative $[\mathbf{1}]^{3-}$: (**a**) at 67 K; (**b**) at 10 K. Data – black trace; Fit – orange trace.

Table S5 Parameters extracted from fit of 10 K X-band EPR data for $[Mo_6O_{19}]^{3-}$ and $[\mathbf{1}]^{3-}$.

	g_x	g_y	g_z	A_x / MHz	A_y / MHz	A_z / MHz	Isotropic linewidth / G
$[Mo_6O_{19}]^{3-}$	1.9123 ± 0.0055	1.9258 ± 0.0059	1.9258 ± 0.0073	209.30	99.91	93.46	1.0
$[\mathbf{1}]^{3-}$	1.9138 ± 0.0058	1.9254 ± 0.0058	1.9266 ± 0.0100	210.17	98.02	89.14	1.5

The spectra (Figure S7) show minimal difference between $[Mo_6O_{19}]^{3-}$ and $[\mathbf{1}]^{3-}$. At 10 K, the shift in the peak at *ca.* 3520 G in $[\mathbf{1}]^{3-}$ towards lower field strength compared to the underivatized cluster is consistent with donation of electron density from the arylimido ligand, to the POM core. The increased linewidth (Table S5) is potentially consistent with weak coupling to the arylimido N, but there is no evidence of a strong interaction between the electron and nitrogen nuclear spin, consistent with the DFT calculated SOMO based predominantly on the other side of the $\{Mo_6\}$ unit to the imido group (Figure 3, main paper).

Hyper-Rayleigh Scattering (HRS)

Measurement of Oxidised (2^-) State HRS. General details of the hyper-Rayleigh scattering (HRS) experiment have been discussed elsewhere,[31] and the experimental procedure and data analysis protocol used for the fs measurements used in this study were as previously described.[32] Measurements were carried out using dilute (*ca.* 10^{-5} M) filtered (Millipore, 0.45 μm) acetonitrile solutions, such that self-absorption of the SHG signal was negligible, verified by the linear relation between signal and concentration. The 1064 nm source was a Spectra-Physics InSight® DS+ laser (1W average power, sub-100 fs pulses, 80 MHz). In this setup, the collection optics are coupled to a spectrograph (model Bruker 500is/sm), together with an EMCCD camera (Andor Solis model iXon Ultra 897). Correction for multiphoton induced fluorescence was done by subtracting the broad MPF background signal from the narrow HRS peak (FWHM ± 9 nm). The high accuracy of this setup enables us to use the solvent as an internal reference (acetonitrile, $\beta_{HRS,1064} = 0.258 \times 10^{-30}$ esu; $\beta_{zzz,1064} = 0.623 \times 10^{-30}$ esu).[33]

In-situ Electrochemically Switched HRS. Electrochemically switched HRS measurements were performed in a custom-designed two compartment cell with the working compartment constructed from a 1 cm path length Pyrex fluorescence cuvette (Figure S8). This preserved three flat, optical quality glass sides enabling collection of scattered SH light at 90° to the incident 1064 nm beam. To minimise potential drift the Ag wire reference electrode was introduced to the working compartment jacketed in a glass tube and fritted disk. The counter electrode was a Pt wire coil and working electrode a Pt gauze, stirring was applied using a magnetic stirrer in the working compartment. The analyte concentration was 10^{-4} M in 0.3 M NBu_4BF_4, higher than for other HRS measurements, for electrochemical performance and to ensure an adequate difference between the sample and solvent/electrolyte baseline.

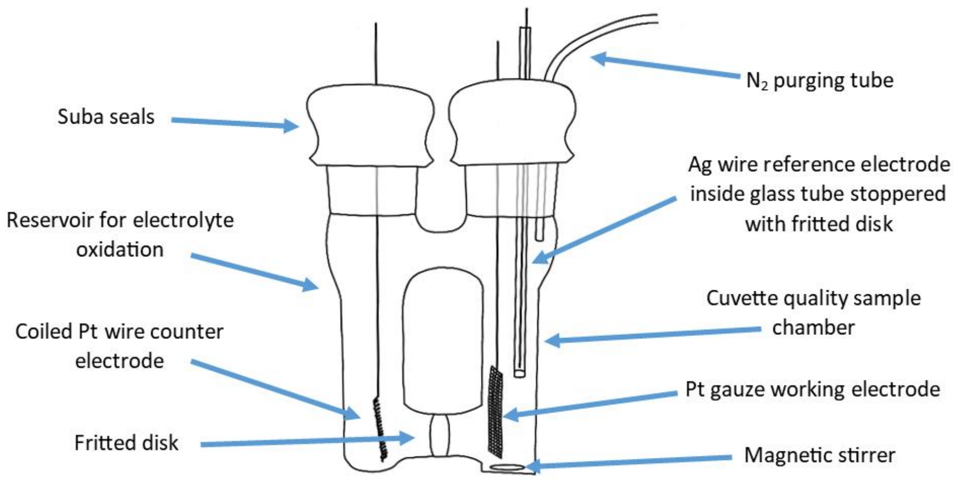

Figure S8 Schematic of the cell used for electrochemically switched HRS.

HRS signal was recorded every 5 seconds, with 10 measurements taken before applying potential to establish a baseline value for the oxidised states. Then a reductive electrochemical potential of -1.3 V vs Ag was applied using a Princeton Applied Research Parstat 2273. To achieve a complete switch-off, potential was applied for 400 seconds before re-oxidation with a potential of 0 V vs Ag (Figure S9). This cycle time is necessitated by the relatively large sample volume, but serves to further demonstrate the high stability of the reduced state of **1**.

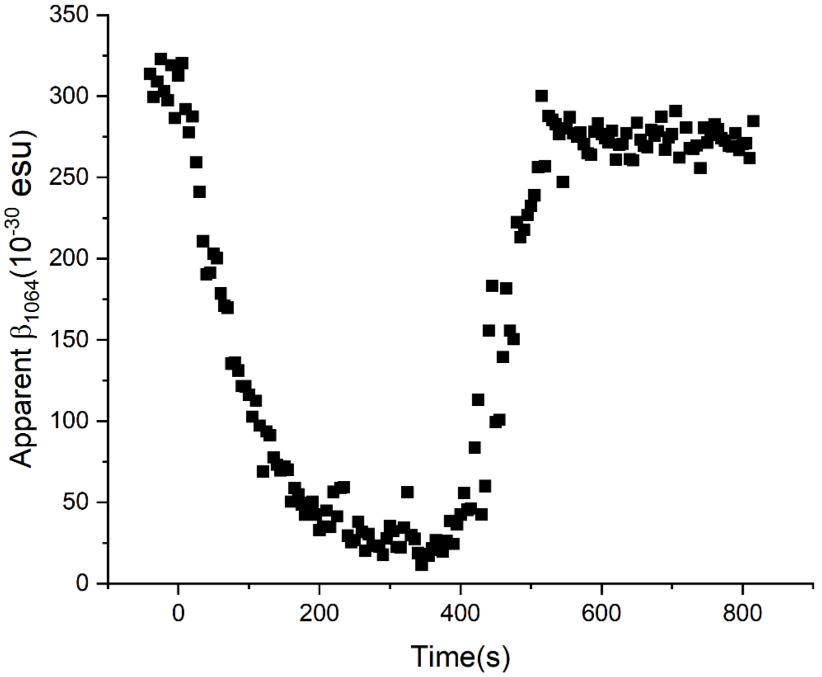

Figure S9 Apparent β_{1064} against time for compound **1** during the initial reduction and reoxidation cycle.

The stability of **1** was further demonstrated over an extended range of reduce/re-oxidise cycles by spectroelectrochemistry, see below:

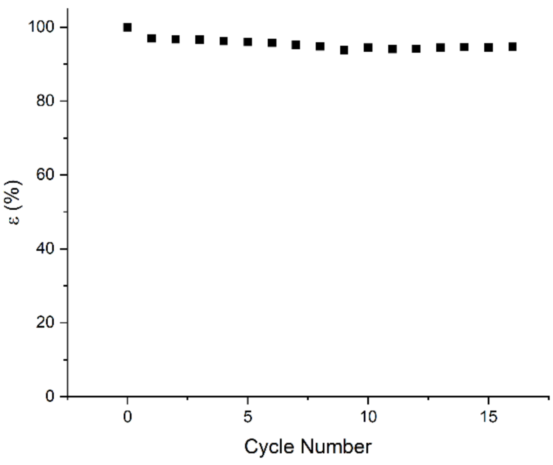

Figure S10 Retention of the absorbance of **1** at 434 nm over 16 full reduce/reoxidise cycles.

Comments on Stability/On-State Signal Loss: It should be noted that as well as a small amount of decomposition of $[\mathbf{1}]^{2-}$, evidenced by initial bulk electrolysis experiments (Figure S10), other electrochemical factors may contribute to the 5 to 10% on-state signal losses seen in Figure 5 (main paper) and above in Figure S10. Bulk electrolyses of reversible oxidations and reductions are intrinsically difficult to drive to completion, as there is an equilibrium between the products. So, while electrolysis may start at 100% $[\mathbf{1}]^{2-}$, reoxidising all the way back to 100% $[\mathbf{1}]^{2-}$ (the "on" state) after the reduction cycle may not be possible without an excessively high overpotential (we have to avoid also oxidising the ligand at +0.3 V vs Fc). Moreover, while the counter electrode is segregated by sintered glass, some slow diffusion of $[\mathbf{1}]^{2-/3-}$ across it is likely to occur. So, during a reduction cycle, the counter will be oxidising solvent/electrolyte to provide electrons to reduce $[\mathbf{1}]^{2-}$, but a more accessible source of electrons will be the ligand oxidation of $[\mathbf{1}]^{2-}$ or even $[\mathbf{1}]^{3-}$ itself; while during reoxidation it is possible that the counter starts reducing small quantities of $[\mathbf{1}]^{2-}$, preventing completion from being achieved. The fact that signal loss stops after a few cycles in both the electrochemistry-HRS experiment, and the spectroelectrochemistry experiment, supports the notion that equilibration and physical factors, rather than chemical degradation, are behind a large part of this signal loss. Overall, the stability seen in these high dilution experiments, that naturally push equilibria towards solvolysis is very encouraging, considering that eventual work with films would involve higher local concentrations of $[\mathbf{1}]^{2-}$, and less access for solvent.

References

1. W. L. F. Armarego, C. L. L. Chai, *Purification of laboratory chemicals*; 6th ed.; Elsevier/Butterworth-Heinemann: Amsterdam; Boston, 2009.

2. W. G. Klemperer, *Inorg. Synth.* **1990**, *27*, 71.

3. F. J. Carver, C. A. Hunter, D. J. Livingstone, J. F. McCabe, E. M. Seward, *Chem. Eur. J.* **2002**, *8*, 2847.

4. I. Bar-Nahum, K. V. Narasimhulu, L.Weiner, R. Neumann, *Inorg Chem.* **2005**, *44*, 4900.

5. *CrysAlisPro* (Version 1.171.40.68a), Rigaku Oxford Diffraction, Rigaku Corporation, Tokyo, Japan, **2019**.

6. G. M. Sheldrick, *Acta Cryst. A*, **2015**, *71*, 3.

7. O. V. Dolomanov, L. J. Bourhis, R. J. Gildea, J. A. K. Howard, H. Puschmann, *J. Appl. Crysallogr.* **2009**, *42*, 339.

8. G. M. Sheldrick, *Acta. Cryst. C*, **2015**, *71*, 3.

9. S. K. Ibrahim, Ph.D Thesis, University of Sussex, **1992**.

10. M.Krejčik, M.Daněk, F.Hartl, *J. Electroanal. Chem.* **1991**, *317*, 179.

11. J.D. Chai and M. Head-Gordon, *Phys. Chem. Chem. Phys.* **2008**, *10*, 6615-6620.

12. A.D. Becke, *J. Chem. Phys*. **1997**, *107*, 8554-8560.

13. R. Krishnan, J.S. Binkley, R. Seeger, J.A. Pople, *J. Chem. Phys.* **1980**, *72*, 650-654.

14. L.E. Roy, P.J. Hay, R.L. Martin, *J. Chem. Theory Comput.* **2008**, *4*, 1029-1031.

15. E. Rtibi, M. Abderrabba, S. Ayadi, B. Champagne, *Inorg. Chem*. **2019**, *58*, 11210-11219.

16. J. Tomasi, B. Mennucci, R. Cammi, *Chem. Rev*. **2005**, *105*, 2999-3094.

17. M.E. Casida, in "Recent advances in Density Functional Theory", edited by D.P. Chong, Singapore, World Scientific (1995), pp 155-192.

18. T. Le Bahers, C. Adamo, I. Ciofini, *J. Chem. Theory Comput.* **2011**, *7*, 2498-2506.

19. S.J.A. Van Gisbergen, J.G. Snijders, E.J. Baerends, *J. Chem. Phys*. **1998**, *109*, 10644-10656.

20. T. Helgaker, S. Coriani, P. Jørgensen, K. Kristensen, J. Olsen, K. Ruud, *Chem. Rev.* **2012**, *112*, 543-631.

21. B. Champagne, P. Beaujean, M. de Wergifosse, M. Cardenuto, V. Liégeois, F. Castet, in Frontiers of Quantum Chemistry, edited by M. Wojcik, H. Nakatsuji, B. Kirtman, Y. Ozaki, Springer, Singapore, 2018, pp 117-138.

22. M. de Wergifosse, B. Champagne, *J. Chem. Phys.* **2011**, *134*, 074113.

23. L.E. Johnson, L.R. Dalton, B.H. Robinson, *Acc. Chem. Res*. **2014**, *47*, 3258-3265.

24. K. Garrett, X.A. Sosa Vazquez, S.B. Egri, J. Wilmer, L.E. Johnson, B.H. Robinson, C.M. Isborn, *J. Chem. Theory Comput.* **2014**, *10*, 3821-3831.

25. A. Tuer, S. Krouglov, R. Cisek, D. Tokarz, V. Barzda, *J. Comput. Chem.* **2011**, *32*, 1128-1134.

26. J. Campo, W. Wenseleers, E. Goovaerts, M. Szablewski, G.H Cross. *J. Phys. Chem. C* **2008,** *112*, 287-296.

27. M. de Wergifosse, E. Botek, E. De Meulenaere, K. Clays, B. Champagne, J. *Phys. Chem. B* **2018**, *122*, 4993-5005.

28. M.J. Frisch, G.W. Trucks, H.B. Schlegel, G.E. Scuseria, M.A. Robb, J.R. Cheeseman, G. Scalmani, V. Barone, G.A. Petersson, H. Nakatsuji, X. Li, M. Caricato, A. V Marenich, J. Bloino, B.G. Janesko, R. Gomperts, B. Mennucci, H.P. Hratchian, J. V Ortiz, A.F. Izmaylov, J.L. Sonnenberg, D. Williams-Young, F. Ding, F. Lipparini, F. Egidi, J. Goings, B. Peng, A. Petrone, T. Henderson, D. Ranasinghe, V.G. Zakrzewski, J. Gao, N. Rega, G. Zheng, W. Liang, M. Hada, M. Ehara, K. Toyota, R. Fukuda, J. Hasegawa, M. Ishida, T. Nakajima, Y. Honda, O. Kitao, H. Nakai, T. Vreven, K. Throssell, J.A. Montgomery Jr., J.E. Peralta, F. Ogliaro, M.J. Bearpark, J.J. Heyd, E.N. Brothers, K.N. Kudin, V.N. Staroverov, T.A. Keith, R. Kobayashi, J. Normand, K. Raghavachari, A.P. Rendell, J.C. Burant, S.S. Iyengar, J. Tomasi, M. Cossi, J.M. Millam, M. Klene, C. Adamo, R. Cammi, J.W. Ochterski, R.L. Martin, K. Morokuma, O. Farkas, J.B. Foresman, D.J. Fox, Gaussian16 Revision A.03, **2016**.

29. DrawMol, V. Liégeois, U. Namur, www.unamur.be/drawmol.

30. S. Stoll, A. Schweiger, EasySpin, a comprehensive software package for spectral simulation and analysis in EPR. *J. Magn. Reson.* **2006**, *178*, 42-55.

31. (a) K. Clays, A. Persoons, *Phys. Rev. Lett*. **1991**, *66*, 2980. (b) K. Clays, A. Persoons, *Rev. Sci. Instrum*. **1992**, *63*, 3285. (c) E. Hendrickx, K. Clays, A. Persoons, *Acc. Chem. Res.* **1998**, *31*, 675.

32. (a) G. Olbrechts, R. Strobbe, K. Clays, A. Persoons, *Rev. Sci. Instrum*. **1998**, *69*, 2233. (b) G. Olbrechts, K. Wostyn, K. Clays, A. Persoons, *Optics Lett*. **1999**, *24*, 403. (c) K. Clays, K. Wostyn, G. Olbrechts, A. Persoons, A. Watanabe, K. Nogi, X.-M. Duan, S. Okada, H. Oikawa, H. Nakanishi, H. Vogel, D. Beljonne, J.-L. Brédas, *J. Opt. Soc. Am. B* **2000**, *17*, 256. (d) E. Franz, E. C. Harper, B. J. Coe, P. Zahradnik, K. Clays, I. Asselberghs, *Proc. SPIE–Int. Soc. Opt. Eng*. **2008**, *6999*, 699923-1–699923-11.

33. J. Campo, F. Desmet, W. Wenseleers, E. Goovaerts, *Opt. Express* **2009**, *17*, 4587.
