# Supplementary material for: Electrochemically‐Switched 2nd Order Non‐Linear Optical Response in an Arylimido‐Polyoxometalate with High Contrast and Cyclability
Source: Angew Chem Int Ed Engl. 2022 Dec 27;62(5):e202215537. doi: 10.1002/anie.202215537 (PMC10107823; doi:10.1002/anie.202215537)

checkCIF/PLATON report

Structure factors have been supplied for datablock(s) exp_1113_brh_1_claire_abs

THIS REPORT IS FOR GUIDANCE ONLY. IF USED AS PART OF A REVIEW PROCEDURE FOR PUBLICATION, IT SHOULD NOT REPLACE THE EXPERTISE OF AN EXPERIENCED CRYSTALLOGRAPHIC REFEREE.

No syntax errors found. CIF dictionary Interpreting this report

Datablock: exp_1113_brh_1_claire_abs

Bond precision: C-C = 0.0052 A Wavelength=0.71073

Cell:	a=12.2689(1)	b=24.3437(2)	c=25.2376(2)
	alpha=115.515(1)	beta=102.332(1)	gamma=94.348(1)
Temperature:	100 K		

	Calculated	Reported
Volume	6524.14(12)	6524.13(11)
Space group	P -1	P -1
Hall group	-P 1	-P 1
Moiety formula	C14 H22 Mo6 N2 O18, 2(C16 H36 N), C3 H6 O	C14 H22 Mo6 N2 O18, 2(C16 H36 N), C3 H6 O
Sum formula	C49 H100 Mo6 N4 O19	C49 H100 Mo6 N4 O19
Mr	1624.97	1624.96
Dx,g cm-3	1.654	1.654
Z	4	4
Mu (mm-1)	1.185	1.185
F000	3304.0	3304.0
F000'	3263.84	
h,k,lmax	17,35,36	17,33,35
Nref	41231	33135
Tmin,Tmax	0.823,0.950	0.599,1.000
Tmin'	0.798	

Correction method= # Reported T Limits: Tmin=0.599 Tmax=1.000
AbsCorr = GAUSSIAN

Data completeness= 0.804 Theta(max)= 30.895

R(reflections)= 0.0335(26595) wR2(reflections)= 0.0771(33135)

S = 1.012 Npar= 1483

The following ALERTS were generated. Each ALERT has the format
test-name_ALERT_alert-type_alert-level.
Click on the hyperlinks for more details of the test.

Alert level C

```
PLAT242_ALERT_2_C Low    'MainMol' Ueq as Compared to Neighbors of       C03B Check
PLAT244_ALERT_4_C Low    'Solvent' Ueq as Compared to Neighbors of       C03I Check
PLAT244_ALERT_4_C Low    'Solvent' Ueq as Compared to Neighbors of       C042 Check
PLAT906_ALERT_3_C Large K Value in the Analysis of Variance ......      2.971 Check
PLAT910_ALERT_3_C Missing # of FCF Reflection(s) Below Theta(Min).          8 Note
PLAT911_ALERT_3_C Missing FCF Refl Between Thmin & STh/L=    0.600          4 Report
```

Alert level G

```
PLAT002_ALERT_2_G Number of Distance or Angle Restraints on AtSite          5 Note
PLAT003_ALERT_2_G Number of Uiso or Uij Restrained non-H Atoms ...         10 Report
PLAT083_ALERT_2_G SHELXL Second Parameter in WGHT  Unusually Large       7.23 Why ?
PLAT154_ALERT_1_G The s.u.'s on the Cell Angles are Equal ..(Note)      0.001 Degree
PLAT176_ALERT_4_G The CIF-Embedded .res File Contains SADI Records          2 Report
PLAT177_ALERT_4_G The CIF-Embedded .res File Contains DELU Records          2 Report
PLAT178_ALERT_4_G The CIF-Embedded .res File Contains SIMU Records          1 Report
PLAT232_ALERT_2_G Hirshfeld Test Diff (M-X)  Mo04     --O00E     .        5.1 s.u.
PLAT232_ALERT_2_G Hirshfeld Test Diff (M-X)  Mo04     --O00P     .        8.8 s.u.
PLAT232_ALERT_2_G Hirshfeld Test Diff (M-X)  Mo0C     --O00E     .        7.8 s.u.
PLAT232_ALERT_2_G Hirshfeld Test Diff (M-X)  Mo0C     --O00Y     .        6.2 s.u.
PLAT232_ALERT_2_G Hirshfeld Test Diff (M-X)  Mo03     --O00K     .        6.4 s.u.
PLAT232_ALERT_2_G Hirshfeld Test Diff (M-X)  Mo08     --O00K     .       10.1 s.u.
PLAT232_ALERT_2_G Hirshfeld Test Diff (M-X)  Mo08     --O00U     .        7.0 s.u.
PLAT232_ALERT_2_G Hirshfeld Test Diff (M-X)  Mo09     --O01B     .        5.4 s.u.
PLAT300_ALERT_4_G Atom Site Occupancy of C03S       Constrained at     0.8012 Check
PLAT300_ALERT_4_G Atom Site Occupancy of C049       Constrained at     0.8012 Check
PLAT300_ALERT_4_G Atom Site Occupancy of C1         Constrained at     0.1988 Check
PLAT300_ALERT_4_G Atom Site Occupancy of C0         Constrained at     0.1988 Check
PLAT300_ALERT_4_G Atom Site Occupancy of H8         Constrained at     0.8012 Check
PLAT300_ALERT_4_G Atom Site Occupancy of H11        Constrained at     0.8012 Check
PLAT300_ALERT_4_G Atom Site Occupancy of H39        Constrained at     0.8012 Check
PLAT300_ALERT_4_G Atom Site Occupancy of H52        Constrained at     0.8012 Check
PLAT300_ALERT_4_G Atom Site Occupancy of H04I       Constrained at     0.8012 Check
PLAT300_ALERT_4_G Atom Site Occupancy of H04J       Constrained at     0.8012 Check
PLAT300_ALERT_4_G Atom Site Occupancy of H04K       Constrained at     0.8012 Check
PLAT300_ALERT_4_G Atom Site Occupancy of H1A        Constrained at     0.1988 Check
PLAT300_ALERT_4_G Atom Site Occupancy of H1B        Constrained at     0.1988 Check
PLAT300_ALERT_4_G Atom Site Occupancy of H40        Constrained at     0.1988 Check
PLAT300_ALERT_4_G Atom Site Occupancy of H41        Constrained at     0.1988 Check
PLAT300_ALERT_4_G Atom Site Occupancy of H0A        Constrained at     0.1988 Check
PLAT300_ALERT_4_G Atom Site Occupancy of H0B        Constrained at     0.1988 Check
PLAT300_ALERT_4_G Atom Site Occupancy of H0C        Constrained at     0.1988 Check
PLAT302_ALERT_4_G Anion/Solvent/Minor-Residue Disorder (Resd  3  )       18% Note
PLAT302_ALERT_4_G Anion/Solvent/Minor-Residue Disorder (Resd  4  )       12% Note
PLAT380_ALERT_4_G Incorrectly? Oriented X(sp2)-Methyl Moiety .....       C02P Check
PLAT380_ALERT_4_G Incorrectly? Oriented X(sp2)-Methyl Moiety .....       C03O Check
PLAT380_ALERT_4_G Incorrectly? Oriented X(sp2)-Methyl Moiety .....       C03P Check
PLAT410_ALERT_2_G Short Intra H...H Contact  H01E     ..Ho3A     .       2.13 Ang.
                                                      x,y,z  =     1_555 Check
PLAT410_ALERT_2_G Short Intra H...H Contact  H01G     ..HaA      .       2.11 Ang.
```

```
                                              x,y,z  =      1_555 Check
PLAT410_ALERT_2_G Short Intra H...H Contact  H01I     ..Ho3B     .       1.75 Ang.
                                              x,y,z  =      1_555 Check
PLAT410_ALERT_2_G Short Intra H...H Contact  H39      ..H02W     .       2.04 Ang.
                                              x,y,z  =      1_555 Check
PLAT410_ALERT_2_G Short Intra H...H Contact  H02R     ..H1B      .       1.92 Ang.
                                              x,y,z  =      1_555 Check
PLAT411_ALERT_2_G Short Inter H...H Contact  H27      ..Ho4B     .       2.11 Ang.
                                           -1+x,y,z  =      1_455 Check
PLAT720_ALERT_4_G Number of Unusual/Non-Standard Labels ..........        308 Note
PLAT794_ALERT_5_G Tentative Bond Valency for Mo01      (VI)      .       6.02 Info
PLAT794_ALERT_5_G Tentative Bond Valency for Mo02      (VI)      .       6.03 Info
PLAT794_ALERT_5_G Tentative Bond Valency for Mo03      (VI)      .       5.93 Info
PLAT794_ALERT_5_G Tentative Bond Valency for Mo04      (VI)      .       5.88 Info
PLAT794_ALERT_5_G Tentative Bond Valency for Mo05      (VI)      .       5.99 Info
PLAT794_ALERT_5_G Tentative Bond Valency for Mo06      (VI)      .       5.93 Info
PLAT794_ALERT_5_G Tentative Bond Valency for Mo07      (VI)      .       5.95 Info
PLAT794_ALERT_5_G Tentative Bond Valency for Mo08      (VI)      .       5.95 Info
PLAT794_ALERT_5_G Tentative Bond Valency for Mo0A      (VI)      .       5.95 Info
PLAT794_ALERT_5_G Tentative Bond Valency for Mo0B      (VI)      .       5.97 Info
PLAT794_ALERT_5_G Tentative Bond Valency for Mo0C      (VI)      .       5.92 Info
PLAT860_ALERT_3_G Number of Least-Squares Restraints .............         48 Note
PLAT912_ALERT_4_G Missing # of FCF Reflections Above STh/L=  0.600       7913 Note
PLAT941_ALERT_3_G Average HKL Measurement Multiplicity ...........        3.3 Low
PLAT951_ALERT_5_G Calculated (ThMax) and CIF-Reported Kmax Differ           2 Units
PLAT957_ALERT_1_G Calculated (ThMax) and Actual (FCF) Kmax Differ           2 Units
PLAT978_ALERT_2_G Number C-C Bonds with Positive Residual Density.          0 Info
```

---

   0 **ALERT level A** = Most likely a serious problem - resolve or explain
   0 **ALERT level B** = A potentially serious problem, consider carefully
   6 **ALERT level C** = Check. Ensure it is not caused by an omission or oversight
  62 **ALERT level G** = General information/check it is not something unexpected

```
   2 ALERT type 1 CIF construction/syntax error, inconsistent or missing data
  19 ALERT type 2 Indicator that the structure model may be wrong or deficient
   5 ALERT type 3 Indicator that the structure quality may be low
  30 ALERT type 4 Improvement, methodology, query or suggestion
  12 ALERT type 5 Informative message, check
```

It is advisable to attempt to resolve as many as possible of the alerts in all categories. Often the minor alerts point to easily fixed oversights, errors and omissions in your CIF or refinement strategy, so attention to these fine details can be worthwhile. In order to resolve some of the more serious problems it may be necessary to carry out additional measurements or structure refinements. However, the purpose of your study may justify the reported deviations and the more serious of these should normally be commented upon in the discussion or experimental section of a paper or in the "special_details" fields of the CIF. checkCIF was carefully designed to identify outliers and unusual parameters, but every test has its limitations and alerts that are not important in a particular case may appear. Conversely, the absence of alerts does not guarantee there are no aspects of the results needing attention. It is up to the individual to critically assess their own results and, if necessary, seek expert advice.

Publication of your CIF in IUCr journals

A basic structural check has been run on your CIF. These basic checks will be run on all CIFs submitted for publication in IUCr journals (*Acta Crystallographica*, *Journal of Applied Crystallography*, *Journal of Synchrotron Radiation*); however, if you intend to submit to *Acta Crystallographica Section C* or *E* or *IUCrData*, you should make sure that full publication checks are run on the final version of your CIF prior to submission.

Publication of your CIF in other journals

Please refer to the *Notes for Authors* of the relevant journal for any special instructions relating to CIF submission.

PLATON version of 12/09/2022; check.def file version of 09/08/2022

Datablock exp_1113_brh_1_claire_abs - ellipsoid plot

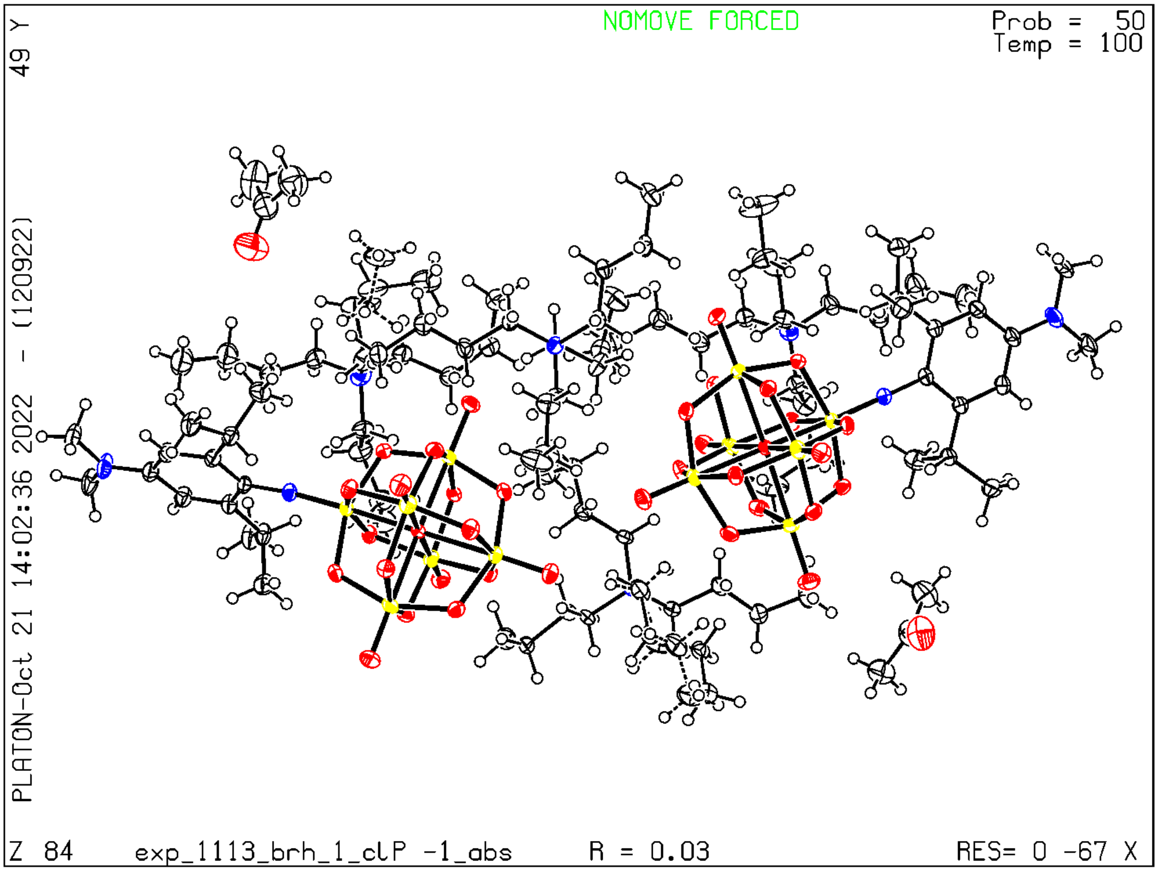

Supplement: Supplementary file 3 — Supporting Information [file ANIE-62-0-s002.pdf]
